# *Odontonia plurellicola* sp. n. and *Odontonia bagginsi* sp. n., two new ascidian-associated shrimp from Ternate and Tidore, Indonesia, with a phylogenetic reconstruction of the genus (Crustacea, Decapoda, Palaemonidae)

**DOI:** 10.3897/zookeys.765.25277

**Published:** 2018-06-07

**Authors:** Werner de Gier, Charles H.J.M. Fransen

**Affiliations:** 1 Research group Taxonomy & Systematics, Naturalis Biodiversity Center, Vondellaan 55, 2332 AA Leiden, The Netherlands

**Keywords:** Ascidians, Caridea, new species, *Odontonia*, Palaemonidae, symbiosis, Ternate, Tidore

## Abstract

Two new species of palaemonid shrimp associated with ascidian hosts, *Odontonia bagginsi* **sp. n.** from Tidore and *Odontonia plurellicola* **sp. n.**, from Ternate, Indonesia are described and figured. Through phylogenetic analyses based on both morphological and molecular datasets (mitochondrial Cytochrome c oxidase subunit I gene and the 16S mitochondrial ribosomal gene) of the genus *Odontonia*, the phylogenetic positions of the new species have been reconstructed. Scanning Electron Microscopy has been used to observe additional characters on dactyli of the ambulatory pereiopods. *Odontonia plurellicola* **sp. n.** appears to be more closely related to *O. simplicipes* and *O. seychellensis*, but it differs most notably in the morphology of the rostrum and mouthparts. *Odontonia plurellicola* **sp. n.** appears to be the only *Odontonia* species living inside a phlebobranch ascidian *Plurella* sp. *Odontonia bagginsi* **sp. n.** is closely related to *O. sibogae*, but differs markedly in the abundance of setae on the propodi of the ambulatory pereiopods. In the present paper, *O. maldivensis* Fransen, 2006 is regarded as a junior synonym of *O. rufopunctata* Fransen, 2002 based on both morphological and molecular aspects.

## Introduction

The palaemonid genus *Odontonia* Fransen, 2002, currently contains 7 species (Fransen 2002, De Grave and Fransen 2011): *O. compacta* (Bruce, 1996); *O. katoi* (Kubo, 1940); *O. maldivensis* Fransen, 2006; *O. rufopunctata* Fransen, 2002; *O. seychellensis* Fransen, 2002, *O. sibogae* (Bruce, 1972); and *O. simplicipes* (Bruce, 1996). As far as is known these species are endosymbionts of solitary ascidians (Fransen 2002) and are distributed in the Indo-West Pacific.

In 2009 an expedition was organised by the Naturalis Biodiversity Center (Leiden, the Netherlands) and the Research Center of Oceanography, Indonesian Institute of Sciences (RCO-LIPI) (Jakarta, Indonesia), to the Indonesian islands of Ternate and Tidore (Hoeksema and van der Meij 2010). During the expedition, two *Odontonia* species new to science were collected from ascidian hosts. The species are described and figured. Their phylogenetic relationships with other members of the genus are analysed using both morphological as well as molecular datasets. The host preference is analysed in relation to the phylogenetic relationships within the genus. A key to the species in the genus is provided.

The following abbreviations are used: COI, mitochondrial gene cytochrome c oxidase subunit I; 16S, 16S mitochondrial ribosomal gene; PoCL, post orbital carapace length; RMNH, Naturalis Biodiversity Center, Leiden (formerly Rijksmuseum van Natuurlijke Historie); MZB, Museum Zoologicum Bogoriense, Research Center for Biology, Indonesian Institute of Sciences, Cibenong, Indonesia.

## Materials and methods

Specimens were gathered around Ternate, in the northern Moluccas during an expedition organised from 23 October to 18 November 2009, by the Research Center of Oceanography, Indonesian Institute of Sciences (RCO-LIPI) (Jakarta, Indonesia) and Naturalis Biodiversity Center (NBC) (Leiden, the Netherlands). This expedition was part of the Ekspedisi Widya Nusantara project (E-Win expeditions). Specimens were collected using SCUBA equipment. Live specimens were photographed with a Nikon D80 digital camera and preserved in 70% ethanol.

Specimens were studied and drawn using a dissecting stereomicroscope (Zeiss Discovery.V8) and a compound microscope (Olympus BX53) both provided with a drawing tube. Sketches were traced using 2.5 to 3 mm Sakura Pigma Micron-pens and scanned (Canon Canoscan 9000F) with a resolution of 600 dpi. Details of the third pereiopods were photographed with a Scanning Electron Microscope. Pereiopods were dried using Critical Point Drying-methods (CPD) in a Leica EM CPD300 (located in Biopartner, Leiden, the Netherlands) with the following parameters (standard protocol preservation insects): CO_2_ intake: Auto, Speed slow, Delay 120s; Exchange: Speed 5, Cycles 18; Gas output: Heat medium, Speed slow 100%. The pereiopods were placed on stubs/mounts in pairs of two species, and coated with 20 nm Pl/Pd using a Quorum Q150T S. The dactyli were photographed using a JEOL JSM-S480LV Scanning Electron Microscope (located in the Naturalis Biodiversity Center, Leiden, the Netherlands). Drawings and photographs were edited in Adobe Photoshop (CS6) for better contrast and brightness.

Morphological character state analysis (Appendix I) was based on specimens in the RMNH collection (Table 1) and literature (Bruce 1996, Fransen 2002, Marin and Anker 2008). Two species of the closely related genera *Pontonia* Latreille, 1829 (*P. panamica* Marin & Anker, 2008; *P. pinnophylax* (Otto, 1821) and *Dactylonia* Fransen, 2002 (*D. ascidicola* (Borradaile, 1898)) were included as outgroup in the analyses. A datamatrix was constructed with ordered (Fitch 1971) and unordered (Farris 1970) characters states (Table 2). The analysis was performed using exhaustive search in PAUP 4.0 (Swofford 2003). Trees were viewed in FigTree 1.4.2 (Rambaut 2009).

**Table 1.** Specimens used for SEM photography and 16S & COI DNA analyses (roman numerals can be linked back to the phylogenetic trees (Figs 20, 21)).

Species	Sample location	Host organism	Registration number	GenBank 16S	GenBank COI	SEM
*Odontonia bagginsi* sp. n.	Indonesia, Halmahera, Tidore	Unid. ascidian	MZB Cru 4733		MH257316	×
*Odontonia katoi* (Kubo, 1940)	Indonesia, Bali, Tulamben beach	*Polycarpa aurata*	RMNH.CRUS.D.48689	MH251614		
	Indonesia, SW Sulawesi, Spermonde Archipelago	*Polycarpa aurata*	RMNH.CRUS.D.46701			×
	Indonesia, NE Sulawesi, Bitung	*Polycarpa aurata*	RMNH.CRUS.D. 57295	MH251615		
*Odontonia maldivensis* Fransen, 2006	Maldives, S Malé Atoll	*Polycarpa cryptocarpa*	RMNH.CRUS.D.51001			×
	Maldives, Faafu Atoll, Magoodhoo Island	*Polycarpa cryptocarpa*	RMNH.CRUS.D. 57296	MH251616		
	Maldives, Faafu Atoll, Magoodhoo Island	*Herdmania momus* (?)	RMNH.CRUS.D. 57297	MH251617		
*Odontonia plurellicola* sp. n.	Indonesia, W Halmahera, Ternate	*Plurella* sp.	RMNH.MZB Cru 4734			×
*Odontonia rufopunctata* Fransen, 2002	Indonesia, Halmahera mainland, Tanjung Ratemu	Unid. ascidian	RMNH.CRUS.D.53601		MH257314	
	Indonesia, NE Sulawesi, Bitung	*Polycarpa* sp.	RMNH.CRUS.D. 57298	MH251618	MH257313	
	Indonesia, NE Sulawesi, Bitung	*Polycarpa* sp.	RMNH.CRUS.D. 57299	MH251619		
	Indonesia, NE Sulawesi, Bitung	*Polycarpa* sp.	RMNH.CRUS.D57300	MH251620		
	Indonesia, SW Sulawesi, Spermonde Archipelago	Unid. ascidian	RMNH.CRUS.D.48694			×
*Odontonia seychellensis* Fransen, 2002	Seychelles, E of Mahé	Unid. stolidobranch ascidian *	RMNH.CRUS.D.42762			×
*Odontonia sibogae* (Bruce, 1972)	Indonesia, Bali, Sanur	*Polycarpa* sp.	RMNH.CRUS.D.48691	MH251621		
	Indonesia, Halmahera, Tidore	Unid. ascidian	RMNH.CRUS.D.53558		MH257315	
	Indonesia, Borneo, Sabah	*Polycarpa argentata*	RMNH.CRUS.D.53964		JX185703	
	Indonesia, Ambon, E coast	*Polycarpa* sp.	RMNH.CRUS.D.47581			×
**Outgroup species**:						
*Pontonia panamica* Marin & Anker, 2008	Panama, Playa Venao	*Ascidia* sp.	RMNH.CRUS.D.51825	MH251622	MH257312	
*Pontonia pinnophylax* (Otto, 1821)	Cape Verde Islands, S coast of São Vicente	*Pinna rudis*	RMNH.CRUS.D.42607	KU170692		
	Cape verde Island, São Nicocau	*Pinna rudis*	RMNH.CRUS.D.42608	MH251623		
*Dactylonia ascidicola* (Borradaile, 1898)	Indonesia, Bali, Tulamben area	*Ascidia* sp.	RMNH.CRUS.D.48678	KU170688		
	Indonesia, Borneo, Sabah	*Ascidia* sp.	RMNH.CRUS.D.53793		MH257317	

* The host of *Odontonia seychellensis* was identified by the authors as an ascidian from the order of the Stolidobranchia, using the collected ascidian specimens from RMNH.CRUS.D.42762 and basic identification guides (Rocha 2011).

**Table 2.** Data matrix of morphological characters used for phylogenetic analysis. Ordered characters in bold face, other characters unordered.

		Species											
		Outgroup species			*Odontonia* species								
		*Dactylonia ascidicola*	*Pontonia pinnophylax*	*Pontonia panamica*	*Odontonia bagginsi* sp. n.	*Odontonia compacta*	*Odontonia katoi*	*Odontonia maldivensis*	*Odontonia rufopunctata*	*Odontonia seychellensis*	*Odontonia sibogae*	*Odontonia simplicipes*	*Odontonia plurellicola* sp. n.
**Characters**	1	0	1	1	2	2	2	0	2	2	2	0	2
	2	0	0	0	1	2	2	2	2	1	1	2	1
	3	0	0	0	0	0	0	0	0	0	0	1	0
	4	1	0	0	1	1	1	1	1	2	1	2	2
	5	0	0	0	0	1	1	1	0	1	1	1	1
	6	0	0	0	1	1	1	1	1	1	1	0	1
	**7**	0	0	0	0	0	0	0	0	1	0	1	1
	8	0	0	2	1	0	2	1	0	0	0	2	1
	9	1	0	0	1	0	0	0	2	0	0	0	0
	**10**	1	0	0	0	1	1	2	1	1	0	1	2
	11	0	0	0	1	1	1	0	0	1	1	1	1
	**12**	0	0	0	0	1	1	1	1	1	0	1	2
	**13**	0	0	0	0	1	0	0	0	0	0	1	2
	14	1	0	0	1	0	0	0	1	0	0	0	0
	15	2	0	0	1	1	1	1	1	0	1	1	1
	16	1	0	0	2	2	2	2	2	0	2	2	2
	**17**	1	0	0	0	1	1	2	2	0	1	0	0
	18	0	1	0	1	1	1	1	1	0	1	1	1
	19	0	0	0	0	0	0	1	0	1	0	1	1
	20	0	0	0	0	0	0	0	0	0	0	1	1
	**21**	0	0	0	1	1	1	0	0	0	1	0	0
	**22**	0	1	1	0	2	1	1	1	1	?	1	1
	23	1	0	0	?	1	1	1	1	1	1	?	2
	24	0	0	0	1	?	1	1	1	1	0	2	2

Mitochondrial COI (7 sequences) and 16S (12 sequences) were available for a partial molecular phylogenetic reconstruction of the genus *Odontonia* (Table 1). For extraction and sequencing see Brinkmann and Fransen (2016). The available sequences were edited and aligned using the alignment tools BioEdit 7.0.0 (Hall 1999) and ClustalX 2.1 (Larkin et al. 2007). The best-fitted model was found using JModelTest (Posada 2008) (using both the Akaike Information Criterion and Bayesian Information Criterion). The datasets were analysed using PAUP 4.0 (Swofford 2003) and MrBayes (Ronquist and Huelsenbeck 2003). Trees were made visible and edited in FigTree 1.4.2 (Rambaut 2009) and Adobe Illustrator (CS6).

Host records for *Odontonia* species were assembled from the literature (Fransen 2002, 2006) and the present material. Ascidian host classification is based on Tsagkogeorga et al. (2009).

## Systematics

### Superfamily Palaemonoidea Rafinesque, 1815

#### Family Palaemonidae Rafinesque, 1815

##### Genus Odontonia

Taxon classificationAnimaliaDecapodaPalaemonidae

Fransen, 2002

###### Generic diagnosis.

(modified from Fransen 2002). Small sized shrimp of subcylindrical body form. Rostrum well developed, short, depressed; dorsally unarmed; dorsal carina formed by shallow broad central elevation, bordered by lateral depressions; lateral carinae well developed; ventral margin with or without small subdistal tooth. Carapace smooth; inferior orbital angles broadly rounded; orbit feebly developed; supraorbital, epigastric and hepatic spines absent; antennal spine blunt, rounded, not separated from the inferior orbital angle; anterolateral angle of branchiostegite rounded, not strongly produced. Eye normal, with hemispherical cornea. Antennula normal, ventromedial tooth on basal segment usually large; distolateral tooth of basal segment well developed, reaching distal margin of intermediate segment, anterior margin oblique, not extending beyond distolateral tooth; flagella short, composed of few segments. Antenna with basicerite unarmed; scaphocerite well developed, with distolateral tooth strongly developed, bent inward, more than 0.2 times length of scaphocerite. Epistome unarmed. Corpus of paragnaths with two submedian, oblique non-setose carinae. Second thoracic sternite with anterior margin broadly rounded, not produced. Fourth thoracic sternite with medially developed centrally slightly notched or completely fused lateral plates. Fifth thoracic sternite with broad rectangular, medially blunt, partly fused lateral plates. Mandible robust, without palp, molar process stout, incisor process simple, with or without row of denticles along medioventral border. Maxillula with bilobed palp, lower lacinia small, slender with few simple setae. Maxilla with simple palp; bilobed endite; scaphognathite broad; basal endite with many simple setae on upper and lower lacinia, both laciniae well developed; basal endite shorter than palp. First maxilliped with slender palp, basal and coxal endites partly fused, with few short setae along median margin, not forming basket; exopod with caridean lobe; flagellum broad, densely setose distally; epipod large, oval. Second maxilliped with distinct angle in median margin of basis of endopod; exopod with flagellum well developed, plumose setae distally; epipod a small rounded, curled lobe; without podobranch. Third maxilliped with ischiomerus of endopod partly fused to basis, as broad as penultimate segment; exopod well developed, with plumose setae distally; coxa with oval, lateral plate, without median process, arthrobranch absent. First pereiopods with chela simple. Chelae unequal in size, subequal in form; major and minor chelae with one proximal tooth on dactylus and two on fixed finger, dactylus without median carina; fixed finger without median fossa to receive dactylar tooth when fingers closed; fingers not gaping. Ambulatory pereiopods stout, dactylus simple or biunguiculate, with or without accessory teeth on flexor margin of corpus; flexor margin with scattered setae; unguis with or without distal scales. Abdomen smooth; posterior margins of pleura rounded, posterolateral angle of sixth segment blunt or rounded. Uropod with protopodite feebly acute distally, exopod with distolateral margin with mobile spinule, feebly armed. Telson with two pairs of large submarginal dorsal spines, and two pairs of cuspidate setae, and submedian pair of plumose setae at posterior margin.

###### Type species.

The type species: *Pontonia katoi* Kubo, 1940, by original designation, gender feminine.

###### Generic distribution.

Known from shallow coastal waters of the Indo-West Pacific.

###### Hosts.

Associated with Ascidiacea.

##### Odontonia plurellicola sp. n.

Taxon classificationAnimaliaDecapodaPalaemonidae

http://zoobank.org/CEB25096-4C73-48F3-B4CE-7A207E890B4A

Figs 1 , 2 , 3 , 4 , 5 , 6 , 7 , 15A–D , 16F , 17E

###### Material examined.

**Type series.** 1 ovigerous female (**holotype**), PoCL 1.55 mm (MZB Cru 4734), Tarau, W Halmahera, Ternate, Indonesia, 0°50'30"N, 127°22'38.5"E, shallow area with coral followed by sandy slope with coral gardens, 9 m depth, scuba diving, 2-11-2009; in ascidian *Plurella* sp. (Asc. 68), leg. C.H.J.M. Fransen, photo TER.17.0049 – 76; 1 male, PoCL 1.30 mm, 1 ovigerous female, PoCL. 1.50 mm, 2 non-ovigerous females PoCL 0.90–1.05 mm (paratypes) (RMNH.CRUS.D.53554), same data as holotype.

###### Diagnosis.

Rostrum as long as antennular peduncle, with distoventral tooth. Pterygostomial angle produced. Basal segment of antennular peduncle with distolateral tooth minute, medioventral tooth strong, acute. Distolateral tooth of scaphocerite robust, 0.3 length of lamina. Dactylus of ambulatory pereiopods with flexor margin of corpus with few (usually 3) short teeth but without accessory tooth; unguis without terminal scales. Telson with two pairs of medium sized (approx. 0.17 of telson length) submarginal dorsal spines at 0.20 and 0.54 of telson length.

###### Description.

**Figure 1.**
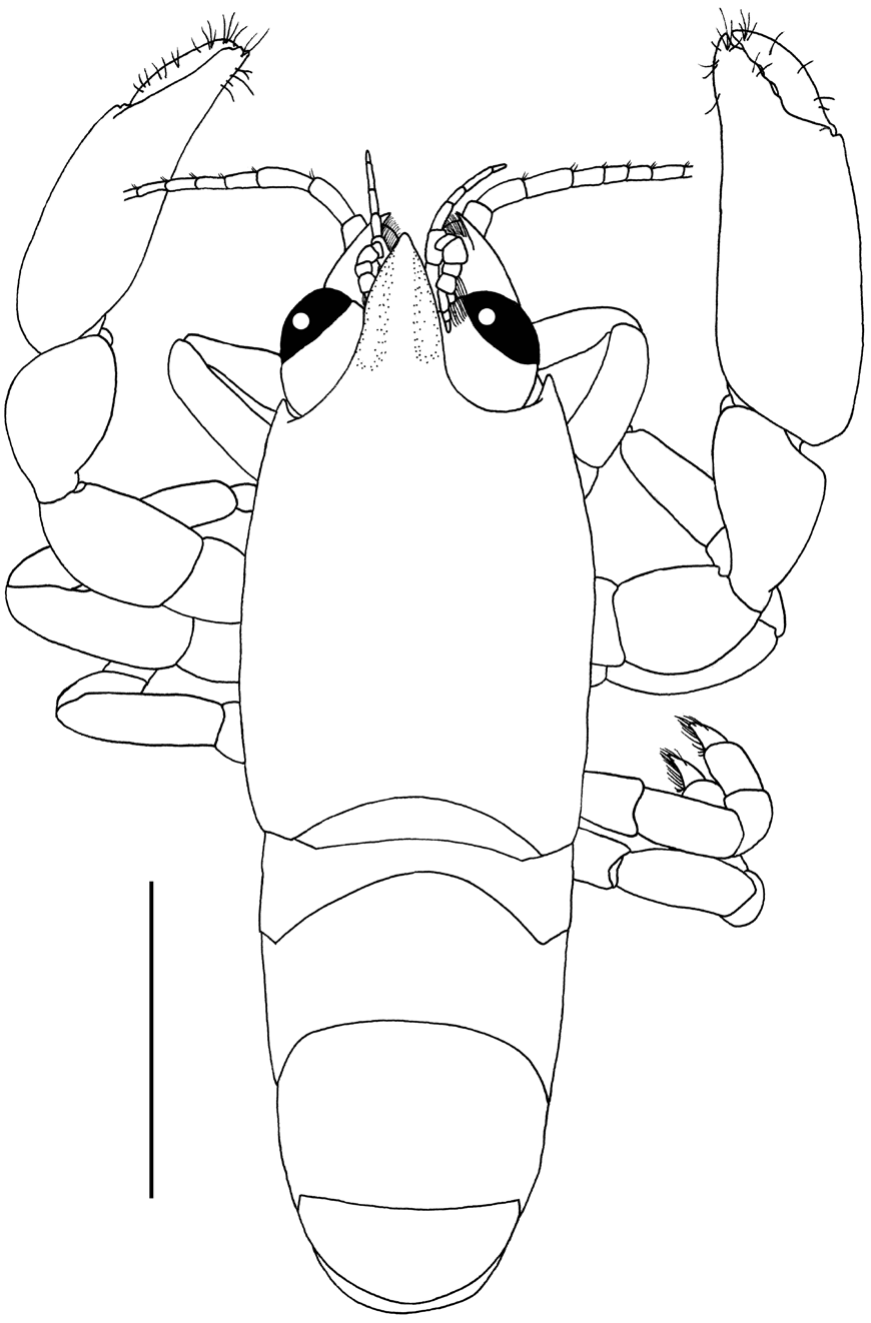
*Odontonia plurellicola* sp. n., habitus, dorsal aspect. Ovigerous female PoCL 1.50 mm (RMNH.CRUS.D.53554). Scale bar: 1 mm.

**Figure 2.**
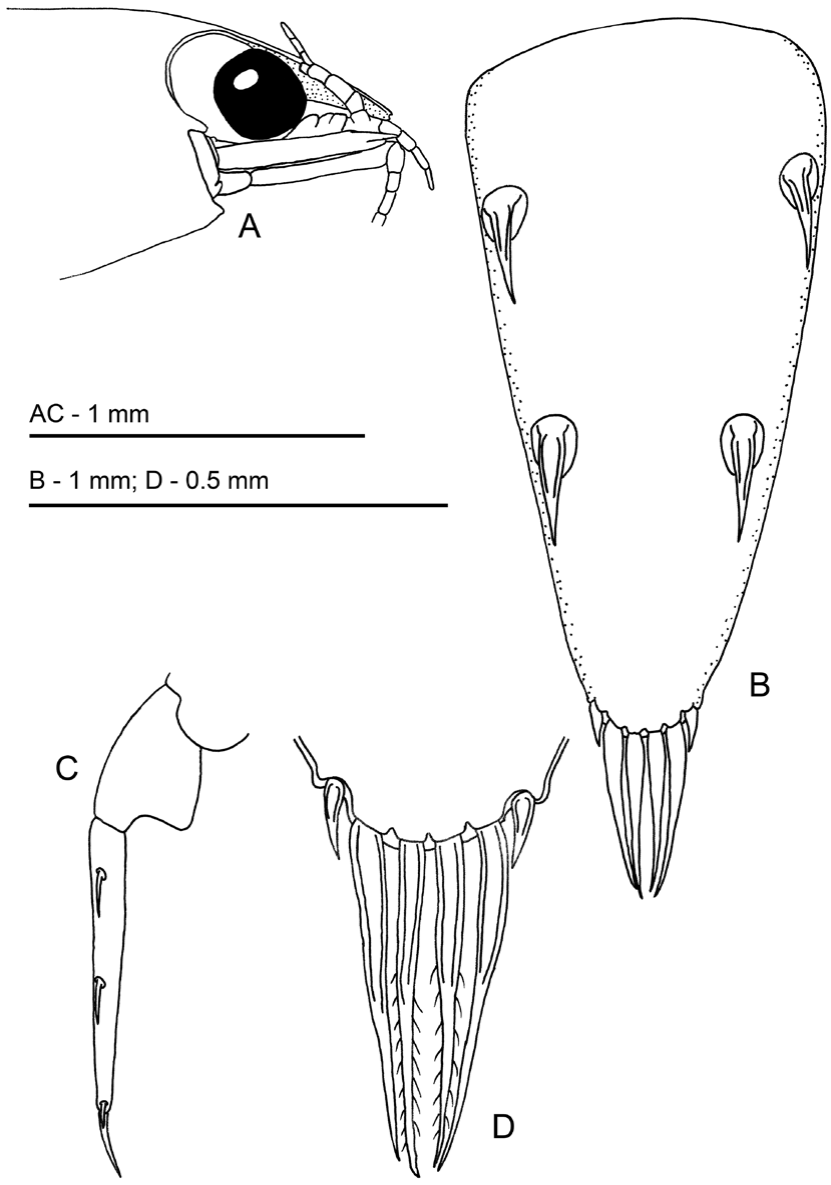
*Odontonia plurellicola* sp. n. ovigerous female PoCL 1.50 mm (RMNH.CRUS.D.53554). **A** anterior appendages, lateral view, setae omitted **B** telson, dorsal view **C** distal part of abdomen, lateral view **D** telson, dorsal view, detail of apex.

Body (Figs 1, 2A) subcylindrical, depressed. Carapace smooth. Rostrum well developed, approx. 0.45 of post-orbital carapace length, as long as antennular peduncle, falling short of distal margin of scaphocerite, approximately 1.8 times longer than diameter of hemispherical cornea, with broad shallow indistinct dorsal carina, with acute lateral carinae, with straight ventral carina; with distal ventral tooth, with distal setae, bluntly acute in dorsal view, broadened at base. Inferior orbital angle not produced, straight. Antennal spine reduced to blunt process. Pterygostomial of carapace straight, anterolateral angle slightly produced, rounded.

Abdomen smooth, sixth segment 1.4 times longer than fifth, 1.4 times wider than long, posterolateral angle blunt, slightly produced, posteroventral angle blunt, not produced; pleura of first five segments broadly rounded.

Telson (Fig. 2B–D) 1.6 times as long as sixth abdominal segment, 2.3 times longer than proximal width; lateral margins almost straight, slightly tapering posteriorly; posterior border without median process; two pairs of medium-sized submarginal dorsal spines at 0.20 and 0.54 of telson length; distal and proximal pair of spines of equal length, 0.17 of telson length; posterior margin with three pairs of spines, lateral spines small, marginal, 0.06 times telson length; submedian spines about as long as intermediate spines, lateral spines 0.23 of submedian and intermediate spines; both intermediate and submedian spines approx. 0.75 of dorsal spine length, but more slender.

Eyestalk short, broader than long, slightly broader than diameter of hemispherical cornea.

**Figure 3.**
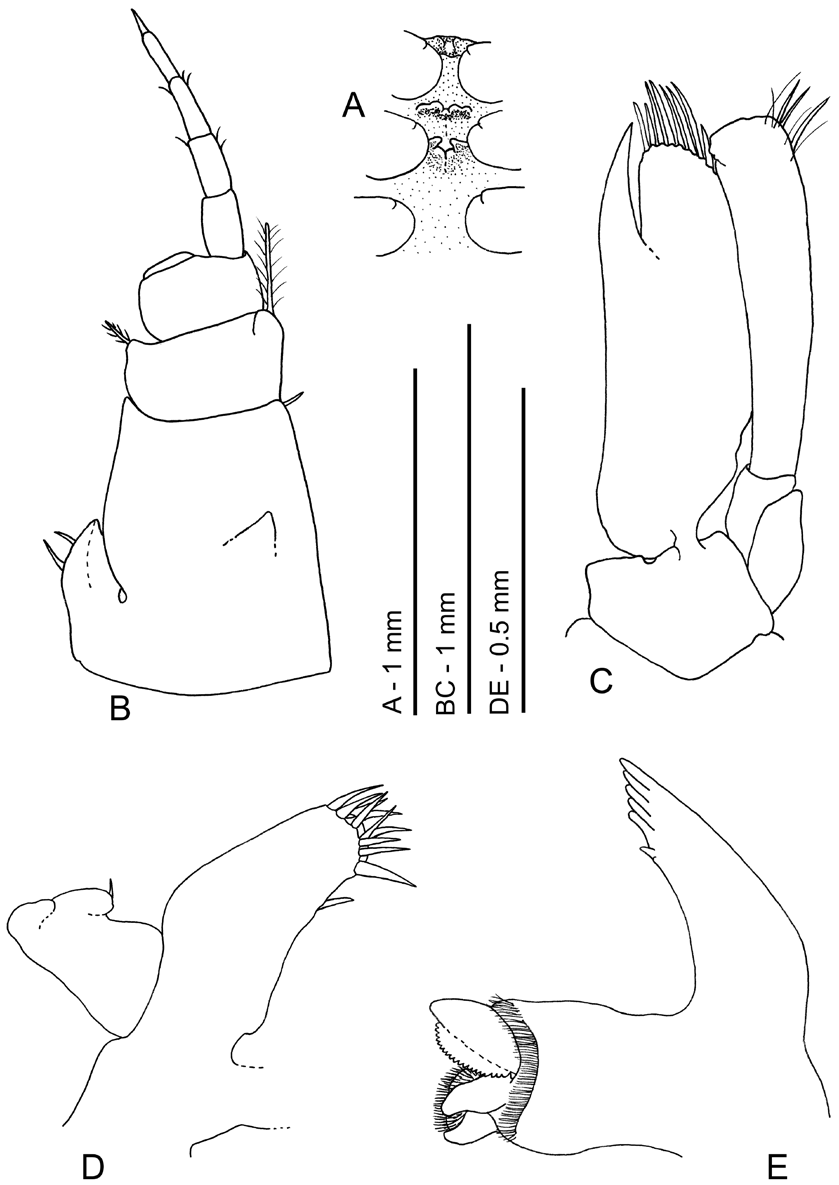
*Odontonia plurellicola* sp. n., ovigerous female PoCL 1.50 mm (RMNH.CRUS.D.53554). **A** second to fifth thoracic sternites **B** antennula, ventral view **C** antenna, ventral view **D** maxillula, ventral view **E** mandible, ventral view.

Antennula (Fig. 3B) with peduncle and flagella short. Basal segment as long as proximal width, with feebly produced distolateral tooth just reaching beyond proximal margin of intermediate segment, anterior margin not developed, oblique; medioventral tooth strongly developed, acute, submarginal, situated halfway basal segment; stylocerite short, reaching halfway basal segment, with acute tip, lateral margin with few plumose setae. Intermediate segment short, broader than long, medial margin with single long distal plumose seta. Distal segment broader than long, upper flagellum short, biramous, with three fused segments; short free ramus one-segmented; longer free ramus with three or four segments. Lower flagellum with four segments; upper ramus carried reflexed beneath lateral rostral carina.

Antenna (Fig. 3C) with basicerite short, laterally unarmed, with large gland tubercle medially; ischiocerite and merocerite normal; carpocerite extending to distal end of distolateral tooth of scaphocerite, rather slender, 4.5 times longer than distal width; flagellum short, slender, nearly as long as postorbital carapace length; scaphocerite with lamina almost twice as long as wide, anterior margin small, rounded, lateral margin broadly convex; distolateral tooth robust, 0.3 length of lamina, reaching beyond lamina, curved medially.

Epistome with rather sharp anterior carina; labrum normal.

Paragnath well developed, alae with broad transverse more or less rectangular distal lobes, and small rounded more or less triangular ventromedial lobes; corpus very short, with shallow median excavation, bordered laterally by non-setose, small, oblique, carinae.

Second thoracic sternite (Fig. 3A) with anterior margin broadly rounded; without median process.

Third thoracic sternite with indistinct shallow lateral carinae.

Fourth thoracic sternite with shallowly developed, medially notched plate formed by the lateral carinae.

Fifth thoracic sternite with well-developed lateral plates with medial broadened deep slit, posteromedial to second pereiopod coxae.

Sixth to eight thoracic sternites unarmed, broadening posteriorly.

Mandible (Fig. 3E) with incisor process with five terminal teeth and one large teeth-like ventromedial denticle; molar process robust, with several blunt teeth, some fringed with setal brushes.

Maxillula (Fig. 3D) with upper lacinia rather small, rectangular with about nine distal spines in two rows, with only few simple setae in distal part; lower lacinia lost in dissection; palp feebly bilobed, larger lobe with small ventral tubercle with single short recurved simple seta.

**Figure 4.**
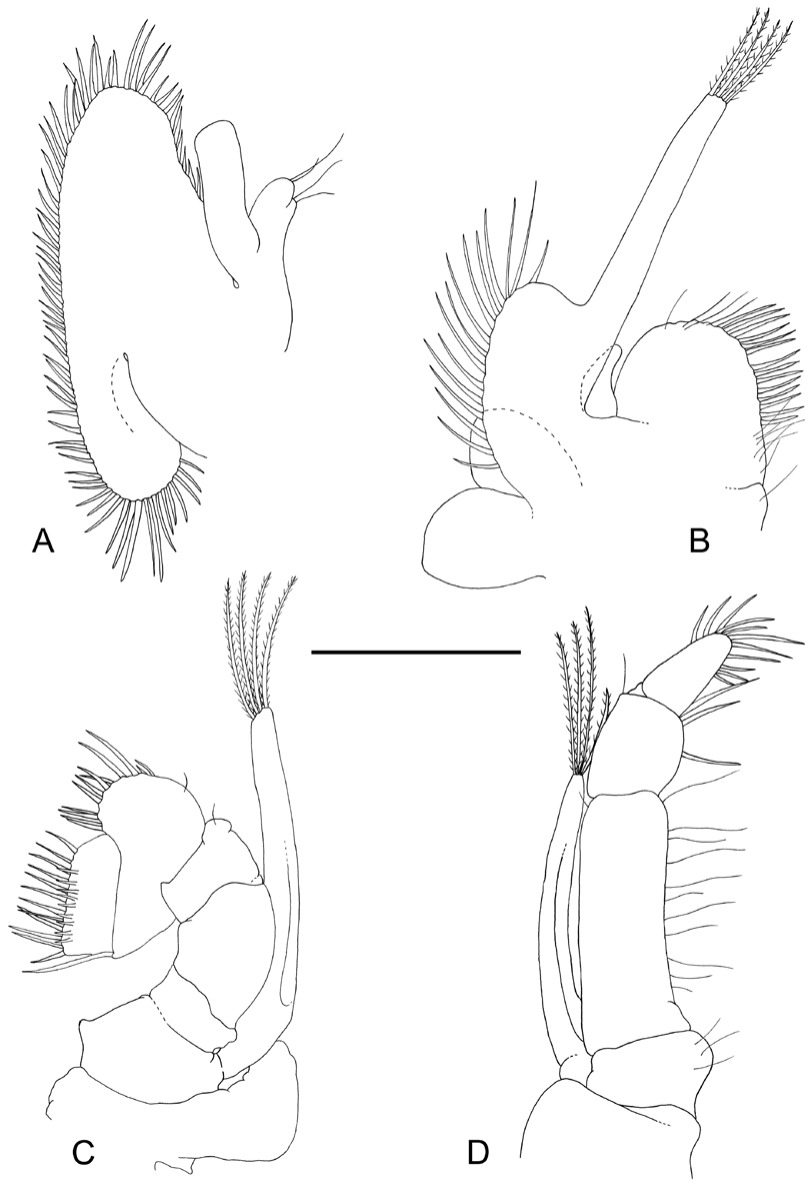
*Odontonia plurellicola* sp. n., ovigerous female PoCL 1.50 mm (RMNH.CRUS.D.53554). **A** maxilla, ventral view **B** first maxilliped, ventral view **C** second maxilliped, dorsal view **D** third maxilliped, ventral view. Scale bar: 1 mm.

Maxilla (Fig. 4A) with basal endite well developed, bilobate, distal and proximal lobe short, distal lobe with two distal seta of unequal length, proximal lobe with two distal setae; coxal endite obsolete, median margin convex, without setae, scaphognathite large, 2.3 times longer than wide, posterior lobe large, 2.3 as long as anterior width, anterior lobe 1.4 times longer than proximal width; palp simple, subquadrate distally, longer than basal endite, not expanding proximally, without row of plumose setae along lateral margin.

First maxilliped (Fig. 4B) with coxal and basal endite partly fused, broad; basal endite fringed with scattered, rather short simple and finely serrulate setae along median and distal margins; coxal endite convex, separated from basal endite, with few simple setae medially; exopod well developed, flagellum with four plumose setae distally; caridean lobe rather small, narrow; epipod bilobate, lobes rounded; palp simple, rather short, non-setose.

Second maxilliped (Fig. 4C) with endopod short, compact; dactylar segment 2.4 times longer than broad, fringed with short, coarsely serrulate, spiniform, and longer curled, finely serrulate setae medially; propodal segment with row of robust spines and few simple setae along expanded distomedian margin; one seta in distal part of ventrolateral margin; carpal segment short, broader than long, unarmed; meral segment without setae, ischial and basal segments partly fused, without setae, basal part angular produced medially; exopod long, with four long plumose setae distally; coxal segment not medially produced, without setae, with proximally expanded epipod laterally.

Third maxilliped (Fig. 4D) short; with ischiomerus distinct from basis, 2.5 times as long as broad, not tapered distally, somewhat flattened, with row of long simple setae along median margin, lateral margin with few simple setae; basal segment medially convex with few simple setae on medial margin; exopod well developed, reaching just beyond distal margin of ischiomerus, with about four long plumose setae in distal part; coxal segment with small median process, with large lateral plate without setae; without arthrobranch; penultimate segment 1.3 times longer than broad, somewhat flattened, with few long finely serrulate setae ventromedially; ultimate segment slightly shorter than penultimate segment, more slender, with groups of long coarsely serrulate setae ventromedially and distally.

**Figure 5.**
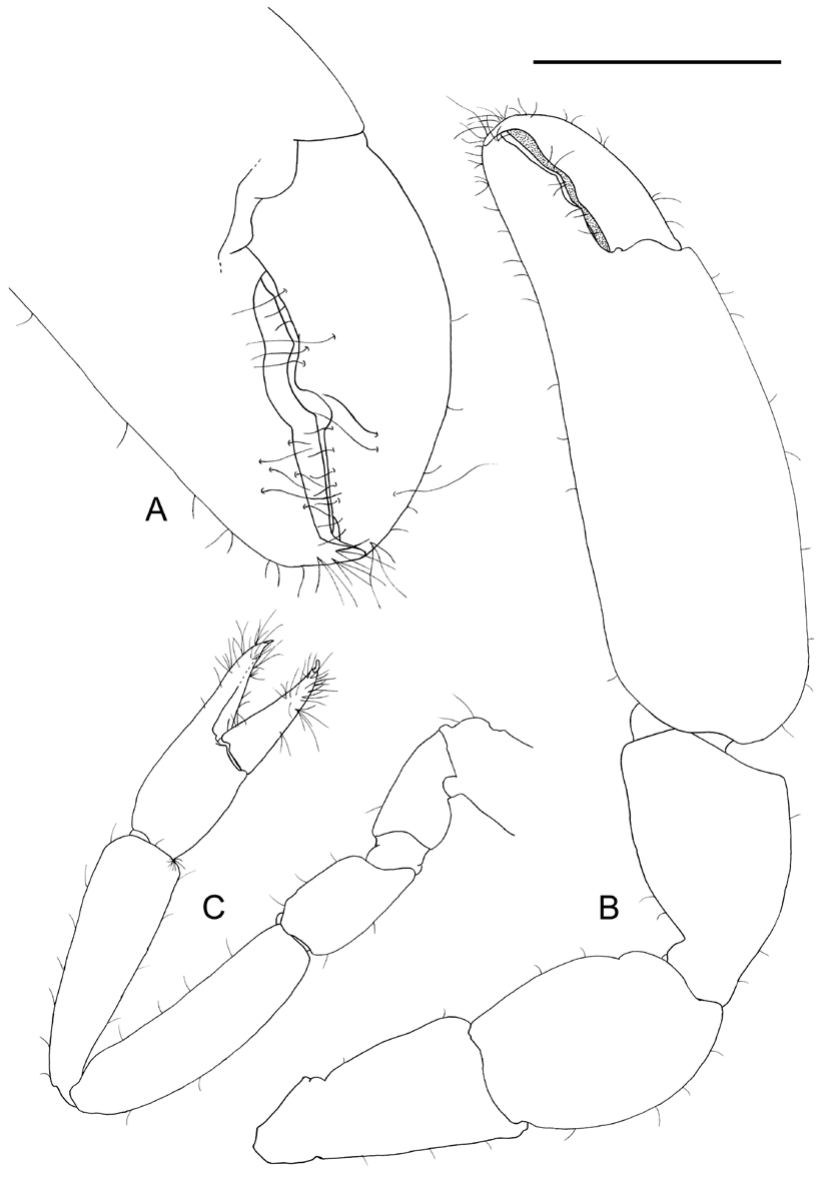
*Odontonia plurellicola* sp. n., ovigerous female PoCL 1.50 mm (RMNH.CRUS.D.53554). **A** major second pereiopod, chela **B** major second pereiopod **C** first pereiopod. Scale bars: 0.5 mm (**A**); 1 mm (**B, C**).

First pereiopod (Fig. 5C) stout, exceeding carpocerite with chela and carpus, chela 2.8 times longer than deep, subcylindrical, slightly compressed; fingers as long as palm, stout, with lateral entire cutting edges, with groups of many serrulate setae, tips slightly hooked, suture of unguis visible; carpo-propodal brush present, serrulate setae in distal part of carpus, no setae in proximal part of palm; carpus 1.2 length of chela, 3.7 times longer than distal width, tapering proximally, unarmed, with medially and laterally few simple setae; merus as long as carpus, 3.7 times longer than central width, somewhat bowed, with few simple setae medially; ischium 0.5 times merus length, slightly expanded medially, with few simple setae medially; basis slightly smaller than ischium, with few simple setae medially; coxa with small ventral lobe with few short simple setae.

Second pereiopods (Fig. 5A–B) subequal, similar. Chela 0.8 times postorbital carapace length in females, major chela about as long as the postorbital carapace length in females, palm smooth, compressed, without carinae, non-setose; fingers with few simple setae in distal part; dactylus 0.39 of palm length, 3.2 times longer than deep, with low, triangular tooth halfway of cutting edge, distal part of cutting edge entire, tip strongly hooked; fixed finger 1.7 times as long as deep, with broad flattened tooth, separated by shallow notch from triangular, small acute tooth at around midpoint of cutting edge, distal part of cutting edge entire, straight, tip strongly hooked; carpus 0.5 of palm length, 1.5 times longer than distal width, strongly tapering proximally; merus as long as carpus, 1.5 times longer than central width, distomedially excavate; ischium slightly shorter than merus, somewhat tapering proximally, with slightly protruded distomedial angle; basis and coxa without special features. Minor cheliped similar, dactylus slightly longer in relation to palm than in major chela; palm less swollen than in major chela.

**Figure 6.**
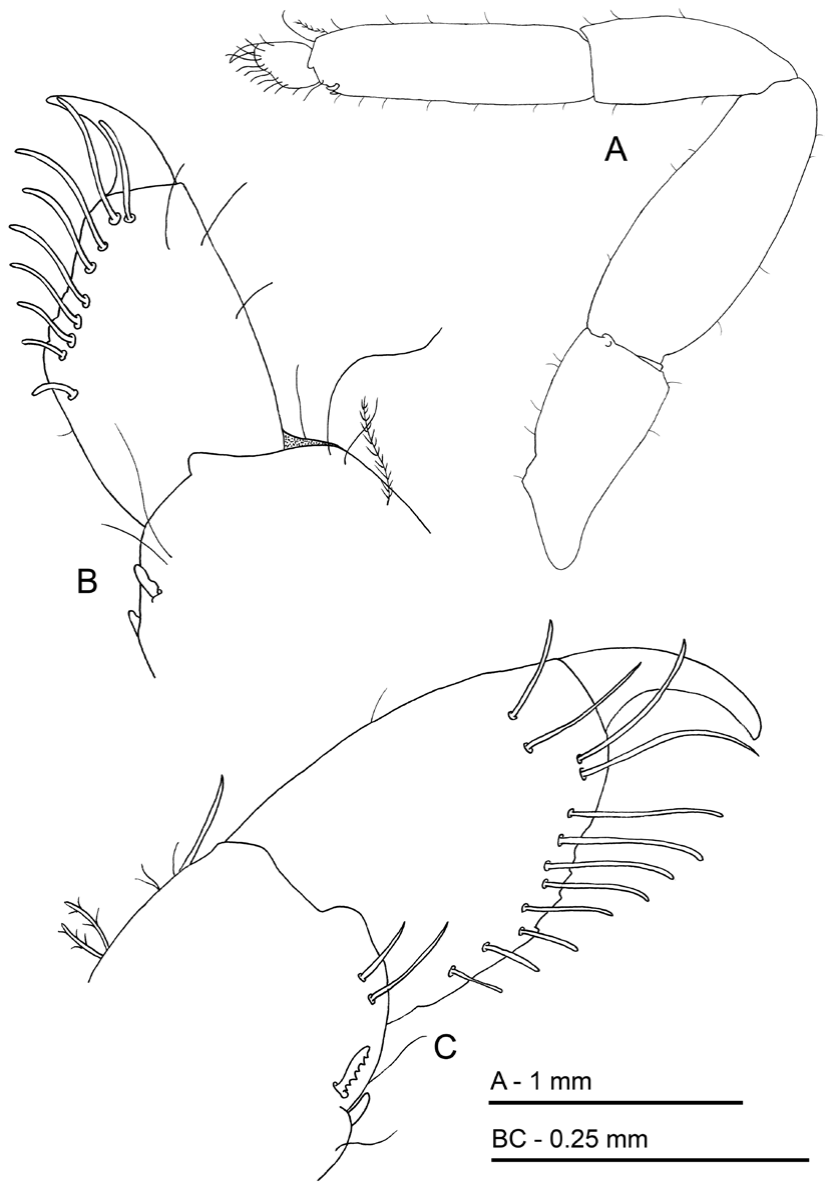
*Odontonia plurellicola* sp. n., ovigerous female PoCL 1.50 mm (RMNH.CRUS.D.53554). **A** third pereiopod **B** dactylus third pereiopod **C** dactylus fifth pereiopod.

Ambulatory pereiopods short, stout. Dactylus of third pereiopod (Fig. 6A, B, 16F, 17E) with corpus compressed, 2.2 times longer than proximal width, with about three small proximal ventrally directing teeth, without accessory tooth, with few simple setae along dorsal margin, with row of simple short setae along flexor margin; unguis long and slender, acute, 0.45 of corpus length, without terminal scales; propodus stout, compressed, 3.7 times length of dactylus, 3.7 times longer than deep, with minute lateral distoventral spinules, and distal ventral spinule with sparse simple setae and one plumose distodorsal setae, carpus 0.6 of propodus length, unarmed, merus subequal to propodus length, 2.2 times longer than central depth, unarmed; ischium 0.6 of merus length, slightly tapering proximally; basis and coxa without special features. Fourth and fifth pereiopods similar, but fifth pereiopod (Fig. 6C) with slightly bigger dactylus, with single lateral pectinate spine.

**Figure 7.**
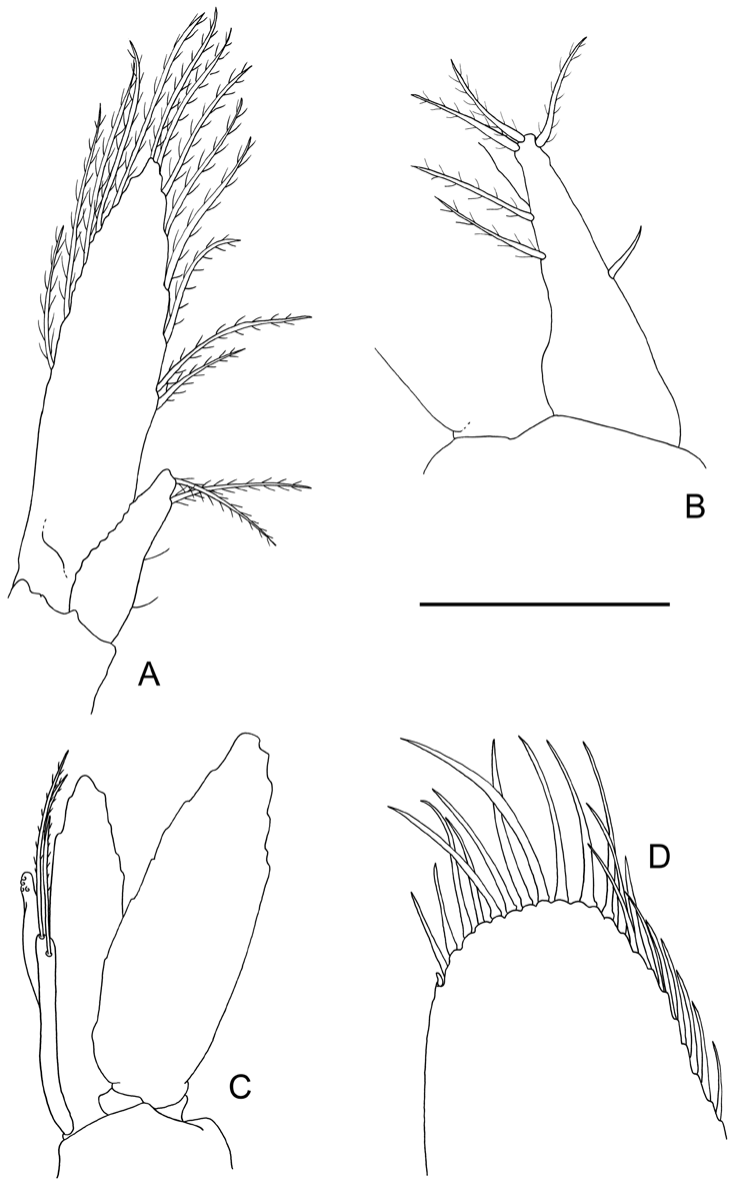
*Odontonia plurellicola* sp. n., ovigerous female PoCL 1.50 mm (**A, D**), male PoCL 1.30 mm (**B, C**) (RMNH.CRUS.D.53554). **A** first pleopod of female with endopod **B** male first pleopod with endopod **C** endopod of second pleopod with appendix masculina and appendix interna **D** detail of left uropod. Scale bars: 1 mm (**A, C, D**); 0.5 mm (**B**).

First pleopod of female (Fig. 7A) with endopod, more than 1/3 as long as exopod, with two long plumose distal setae when ovigerous, with two lateral simple setae. Male first pleopod (Fig. 7B) with endopod about three times as long as proximal width, distinctly tapering distally; median margin straight with single simple setae, with few plumose long setae along lateral and distal margin.

Endopod of second pleopod (Fig. 7C) with appendix masculina about 2/3 length of appendix interna, with three very long setulose setae distally.Uropods (Fig. 7D), with short unarmed protopodite; exopod broad, 2.2 times longer than central width, lateral margin feebly convex, without distolateral tooth, with minute spinule distolaterally; endopod exceeding exopod, about as long as telson, 2.8 times longer than wide.

Number of eggs approximately 11.

###### Size.

This is a small sized species. The maximum PoCL is 1.55 mm in adult females, 1.30 mm in adult males. The minimal PoCL in ovigerous females is 1.50 mm.

###### Colour in life

(Fig. 15A–D). Body with small white chromatophores and scattered larger white spots. Carapace with white chromatophores at base of rostrum and in posterior part, central part without white spots or chromatophores. Laterally covered with white chromatophores and big large white spots. Eyestalks with some big dorsal white spots, cornea with white spots. Antennular peduncle with large white spots distally. Pereiopods without small white chromatophores, with white spots at joints. Palm of chela of second pereiopods with scattered white spots. Abdominal pleura with many small white chromatophores and large white spots dorsally and laterally at fixed distances; in dorsal view, each tergum with a transverse row of white spots anteriorly. First abdominal segment covered with large white spots as fixed distances. Tailfan without chromatophores. Thoracical appendages and tailfan appear to be translucent; carapace, eyestalks, corneas and abdominal segments appear to be darker in colour.

###### Host.

Specimens were found inside a colonial ascidian of the genus *Plurella* Kott, 1973 (Plurellidae, Phlebobranchia).

###### Distribution.

Only known from the type locality.

###### Etymology.

The species is named *plurellicola* after the colonial ascidian genus *Plurella* Kott, 1973 in which it was found.

###### Remarks.

The species resembles *O. simplicipes*, known only by the holotype, in morphological characters. It differs from this species in the length and shape of the rostrum (most notably, *O. simplicipes* has no distal tooth on its rostrum, while *O. plurellicola* bears a small distal tooth), in the size of the ventromedial tooth on the basal segment of the antennular peduncle which is larger in *O. seychellensis* than in the new species, in the distolateral tooth of the basal segment of the antennular peduncle which is well developed in *O. seychellensis* while minute in the new species, in the amount of plumose setae on the three maxillipedes and the antennular peduncle.

Thus far this is the only species of *Odontonia* living inside a colonial ascidian. The ascidian genus *Plurella* has also been recorded as host for *Dactylonia holthuisi* Fransen, 2002, another symbiotic palaemonid shrimp (Fransen 2002, 2006).

##### Odontonia bagginsi sp. n.

Taxon classificationAnimaliaDecapodaPalaemonidae

http://zoobank.org/2AF91392-9595-4D14-9F1B-C9BDB3BDB954

Figs 8 , 9 , 10 , 11 , 12 , 13 , 14 , 15E , 16A , 17A

###### Material examined.

**(i)** Indo-West Pacific: Indonesia. –1 ovigerous female (**holotype**) PoCL 3.4 mm (MZB Cru 4733, ex RMNH.CRUS.D.53559), N of Desa Rum, Tidore, Ternate, Indonesia, 0°44'35.8"N 127°23'6.3"E, 27 m depth, scuba diving, reef consisting primarily of boulders and soft corals; 4-11-2009; in solitary ascidian (Asc. 67), leg. A. Gittenberger, photo TER.17.0136–39.

**Diagnosis.** Rostrum as long as antennular peduncle, with strong distoventral tooth. Pterygostomial angle broadly rounded, produced. Basal segment of antennular peduncle with strong, acute medioventral tooth. Distolateral tooth of scaphocerite robust, 0.4 length of lamina. Dactylus of ambulatory pereiopods with blunt accessory tooth, perpendicular to flexor margin; flexor margin of corpus with strong, acute forward directed proximal tooth and two small denticles between this tooth and accessory tooth; unguis without terminal scales. Telson with two pairs of medium sized (0.13 of telson length) submarginal dorsal spines at 0.15 and 0.48 of telson length.

**Figure 8.**
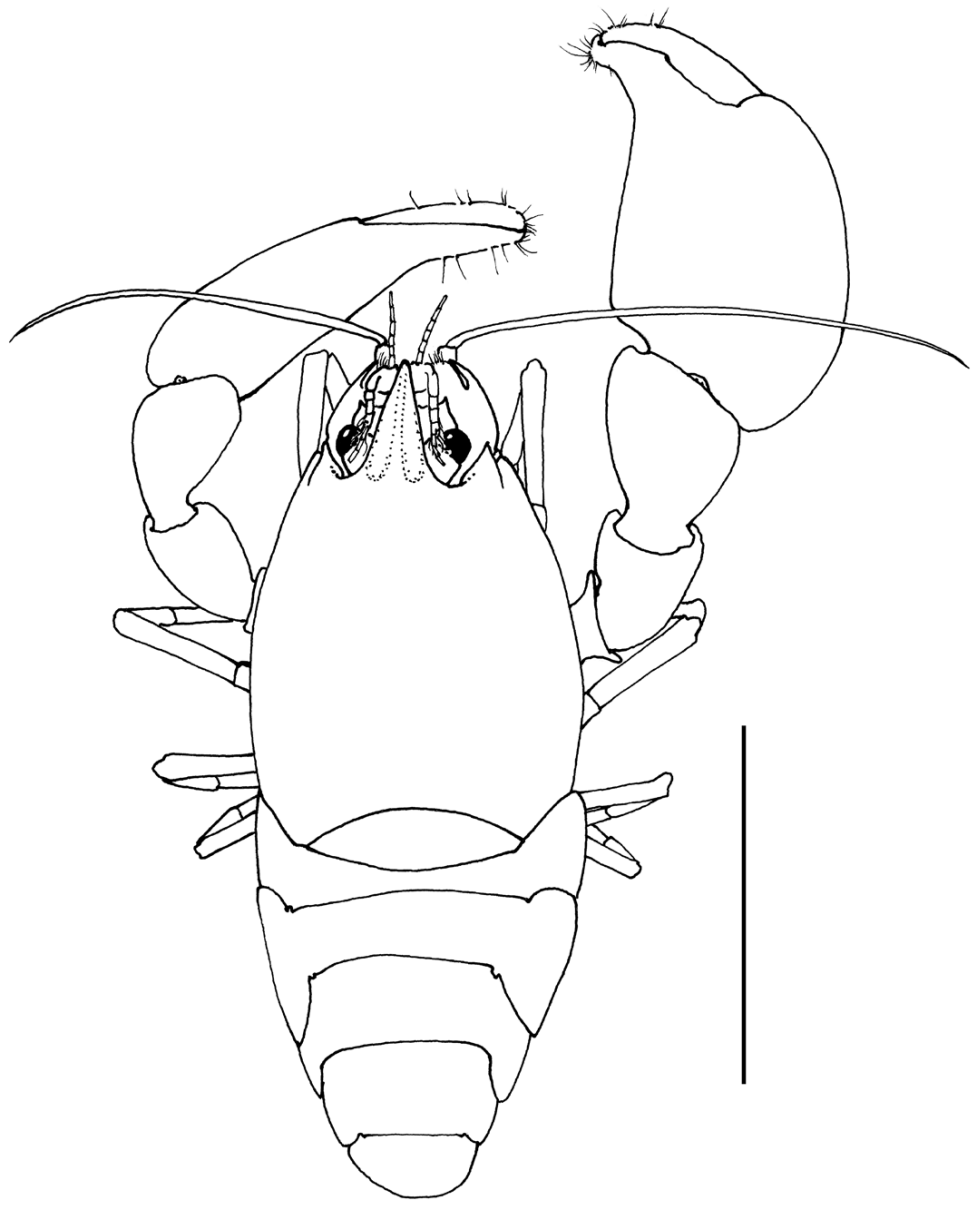
*Odontonia bagginsi* sp. n., ovigerous female PoCL 3.40 mm (MZB Cru 4733). Dorsal aspect. Scale bar: 3 mm.

**Description.** Body (Figs 8, 15E) subcylindrical, depressed. Carapace smooth. Rostrum well developed, 0.38 of post-orbital carapace length, without dorsal teeth, as long as antennular peduncle, reaching distal margin of scaphocerite, approximately 2.6 times longer than diameter of hemispherical cornea, with broad shallow indistinct dorsal carina, with acute lateral carinae, with straight ventral carina; with strong distal ventral tooth just extending beyond apex, with few distal setae, bluntly acute in dorsal view, broadened at base. Inferior orbital angle not produced, straight. Antennal spine reduced to blunt protruding process, not separated by notch from inferior orbital angle. Pterygostomial angle of carapace strongly produced, rounded.

Abdomen smooth, sixth segment 1.3 times longer than fifth, 1.4 times wider than long, posterolateral angle blunt, slightly produced, posteroventral angle blunt, not produced; pleura of first five segments broadly rounded.

Telson (Fig. 10A–B) 1.8 times as long as sixth abdominal segment, 2.0 times longer than proximal width; lateral margins almost straight, slightly convex; posterior border without median process; two pairs of medium-sized submarginal dorsal spines at 0.15 and 0.48 of telson length; distal and proximal pair of dorsal spines of equal length, 0.13 of telson length; posterior margin with three pairs of spines, lateral spines small, submarginal, 0.06 times telson length; submedian spines slightly longer than intermediate spines, lateral spines 0.20 of submedian and intermediate spines; both intermediate and submedian spines 0.7 of dorsal spine length, but more slender.

Eyestalk short, about as long as broad, as broad as diameter of hemispherical cornea.

**Figure 9.**
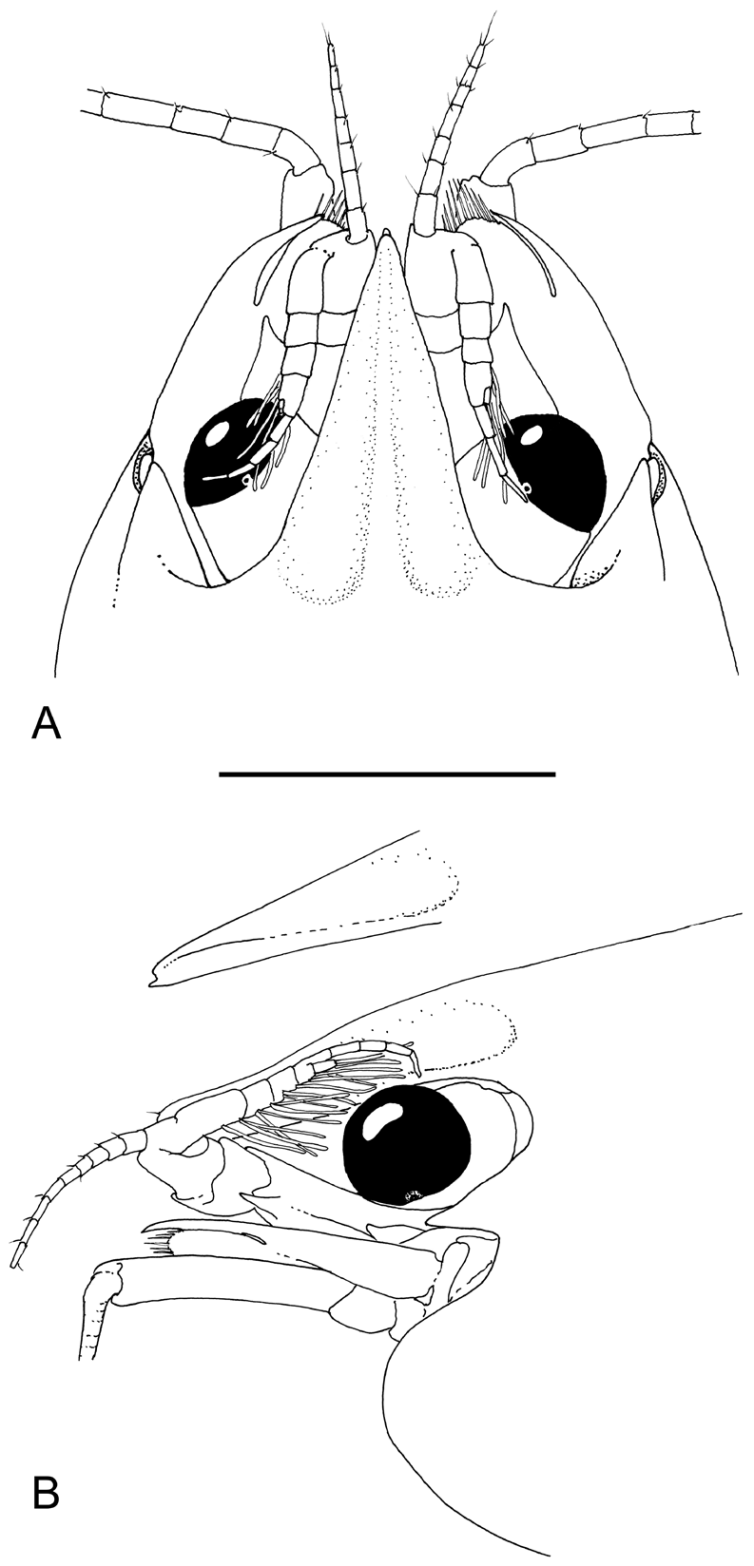
*Odontonia bagginsi* sp. n., ovigerous female PoCL 3.40 mm (MZB Cru 4733). **A** anterior appendages, dorsal view **B** anterior appendages, lateral view. Scale bar: 1 mm.

Antennula (Fig. 9A–B) with peduncle and flagella short. Basal segment as long as proximal width, with acute produced distolateral tooth just falling short of distal margin of intermediate segment, anterior margin not developed, oblique; medioventral tooth strongly developed, acute, submarginal, situated halfway basal segment; stylocerite short, reaching proximal third of basal segment, distally bluntly acute, lateral margin with few plumose setae. Intermediate segment short, broader than long, medial margin with single long distal plumose seta. Distal segment broader than long, upper flagellum short, biramous, with four fused segments; short free ramus one-segmented; longer free ramus with five segments. Lower flagellum with six or seven segments.

Antenna (Fig. 9A–B) with basicerite short, laterally unarmed, with large gland tubercle medially; ischiocerite and merocerite normal; carpocerite extending just beyond distal end of distolateral tooth of scaphocerite, rather slender, 4.2 times longer than distal width; flagellum short, slender, about as long as postorbital carapace length; scaphocerite with lamina about twice as long as wide, anterior margin small, rounded, lateral margin broadly convex; distolateral tooth robust, 0.4 length of lamina (incl. distolateral tooth) reaching beyond lamina, curved medially; incision between distolateral tooth and lamina deep.

Epistome anteriorly broadly rounded; labrum normal, oval.

Paragnath well developed, alae with broad transverse more or less rectangular distal lobes, and small rounded more or less triangular ventromesial lobes; corpus very short, with shallow median excavation, bordered laterally by non-setose, small, oblique, carinae.

Second thoracic sternite with anterior margin broadly rounded; without median process forming round tubercle.

Third thoracic sternite with indistinct shallow lateral carinae.

Fourth thoracic sternite with developed, bluntly triangular medial plate without median notch.

Fifth thoracic sternite with well-developed lateral plates with medial broadened deep slit, posteromedial to second pereiopod coxae.

Sixth to eight thoracic sternites unarmed, broadening posteriorly.

**Figure 10.**
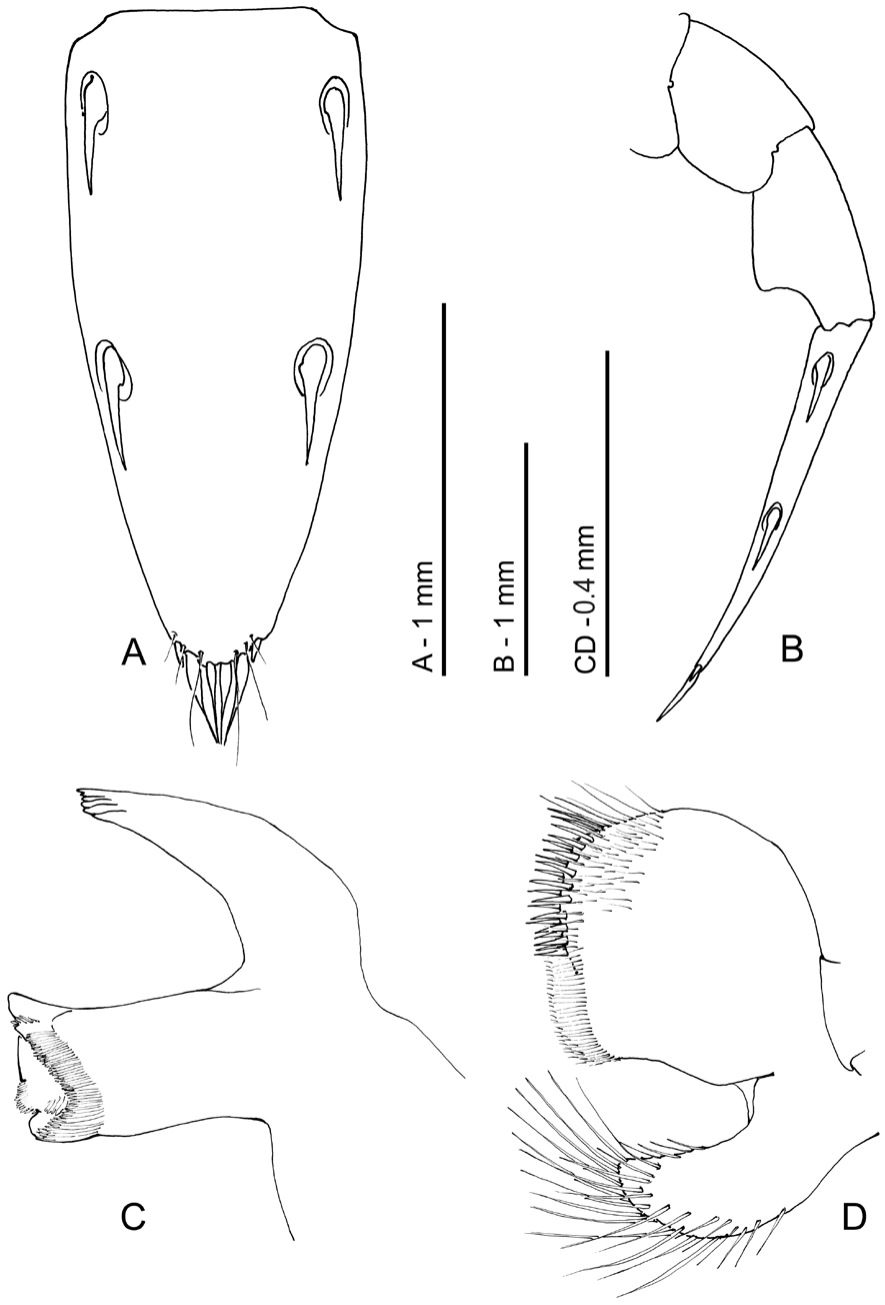
*Odontonia bagginsi* sp. n., ovigerous female PoCL 3.40 mm (MZB Cru 4733). **A** telson, dorsal view **B** telson, lateral view **C** mandible, ventral view **D** maxillula, ventral view.

Mandible (Fig. 10C) with incisor process with five terminal teeth of which larger distalmost is bifid, without ventromedial denticle; molar process robust, with several blunt teeth, some fringed with setal brushed.

Maxillula (Fig. 10D) with upper lacinia broad, rectangular with about 30 spines in two rows in distal half, with many simple setae along entire median margin; lower lacinia slender, acutely pointed upward, with many serrate and simple setae; palp present, but lost in dissection.

**Figure 11.**
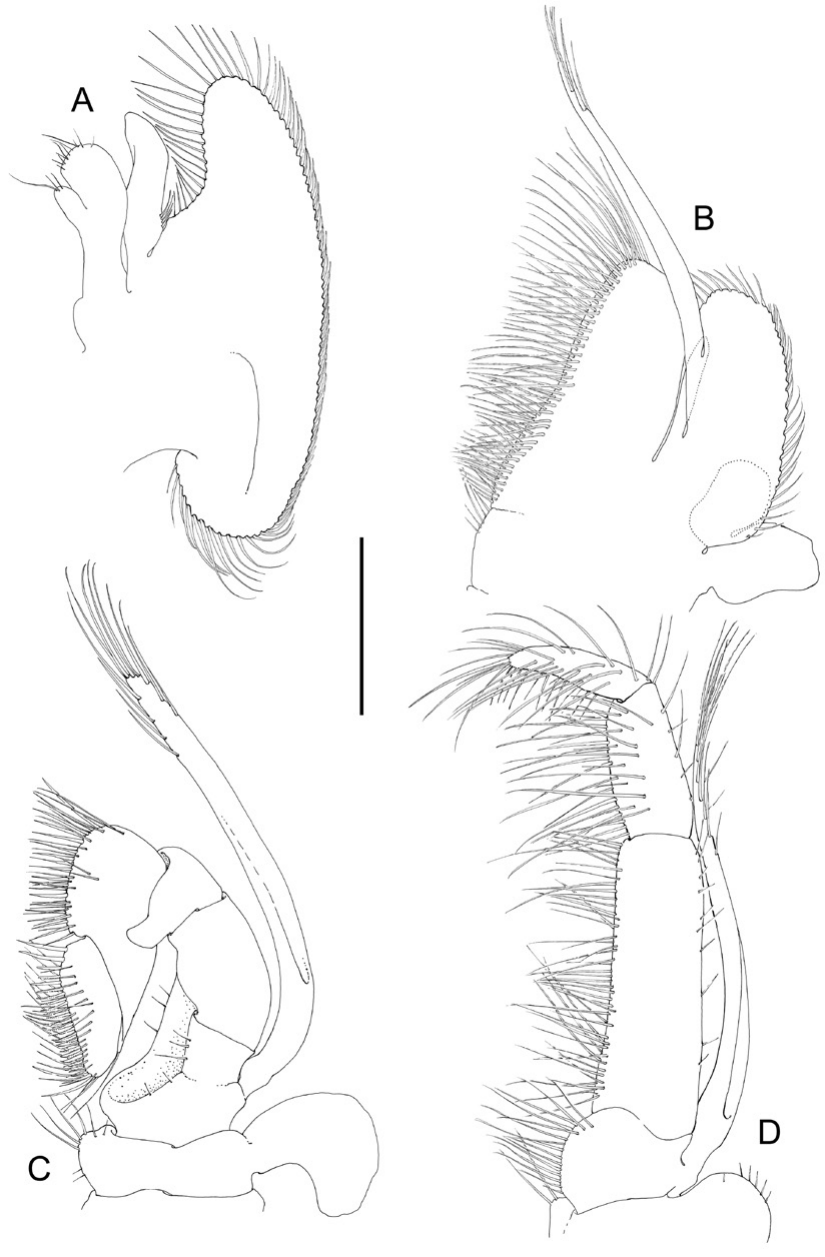
*Odontonia bagginsi* sp. n., ovigerous female PoCL 3.40 mm (MZB Cru 4733). **A** maxilla, ventral view **B** first maxilliped, ventral view **C** second maxilliped, ventral view **D** third maxilliped, ventral view. Scale bar: 0.4 mm.

Maxilla (Fig. 11A) with basal endite well developed, bilobed, distal lobe broad with about 11 short simple distal setae of unequal length, proximal lobe small with 3 distal setae; coxal endite obsolete, median margin convex, without setae; scaphognathite large, 2.5 times longer than wide; palp simple, longer than basal endite, not expanding proximally, with row of about 4 plumose setae along proximolateral margin.

First maxilliped (Fig. 11B) with coxal and basal endite partly fused, broad; basal endite fringed with dense cover of, long simple and finely serrulate setae along median and distal margins; coxal endite convex, feebly demarcated from basal endite, with few simple setae medially; exopod well developed, flagellum with 6 plumose setae distally; caridean lobe rather small, narrow; epipod bilobate, lobes rounded; palp simple, rather short, non-setose.

Second maxilliped (Fig. 11C) with endopod short, compact; dactylar segment 2.6 times times longer than broad, fringed with short, coarsely serrulate, spiniform, and longer curled, finely serrulate setae medially; propodal segment with row of robust spines and few simple setae along expanded distomedian margin; one seta in distal part of ventrolateral margin; carpal segment short, broader than long, unarmed; meral segment without setae, ischial and basal segments almost completely fused, with few short setae, basal part produced medially; exopod long, with 12 long plumose setae in distal part; coxal segment medially produced, with few simple setae, with proximally expanded epipod laterally.

Third maxilliped (Fig. 11D) short; with ischiomerus distinct from basis, 3.2 times as long as broad, not tapered distally, somewhat flattened, with row of long simple setae along median margin, lateral margin with few simple setae; basal segment medially convex with long simple setae on medial margin; exopod well developed, reaching just halfway penultimate segment, with about 16 long plumose setae in distal part; coxal segment with small median process, with large lateral plate with few short setae; without arthrobranch; penultimate segment 2.0 times longer than broad, somewhat flattened, with many finely serrulate setae ventromedially; ultimate segment slightly shorter than penultimate segment, more slender, with groups of long coarsely serrulate setae ventromedially and distally.

**Figure 12.**
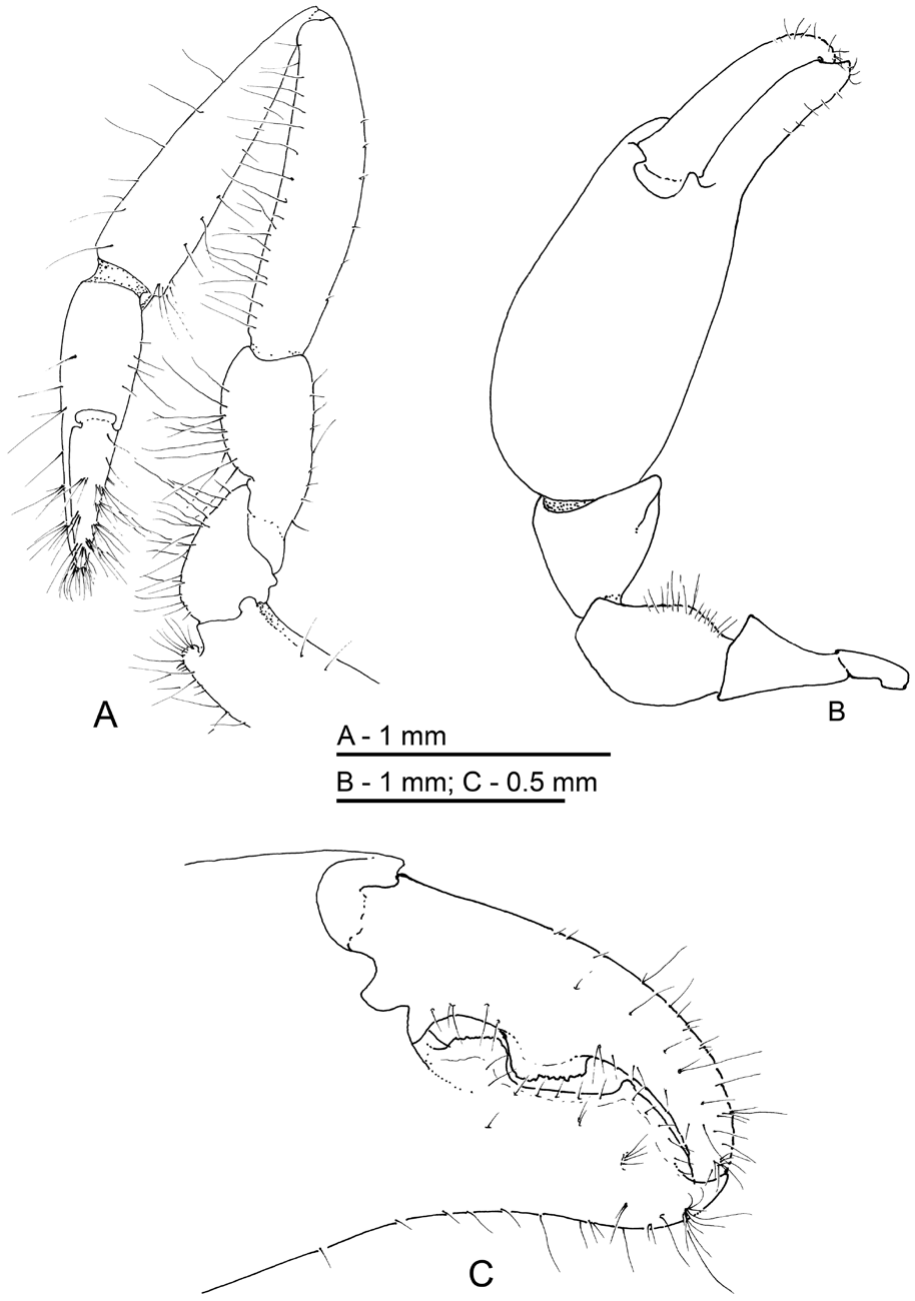
*Odontonia bagginsi* sp. n., ovigerous female PoCL 3.40 mm (MZB Cru 4733). **A** first pereiopod **B** major second pereiopod **C** major second pereiopod, chela.

First pereiopod (Fig. 12A) stout, exceeding carpocerite with chela and carpus, chela 3.2 times longer than deep, subcylindrical, slightly compressed; fingers as long as palm, stout, with lateral entire cutting edges, with groups of many serrulate setae, tips slightly hooked, suture of unguis distinct; carpo-propodal brush present, serrulate setae in distal part of carpus, and proximal part of palm; carpus 1.2 length of chela, 3.9 times longer than distal width, tapering proximally, unarmed, with simple setae medially and laterally; merus as long as carpus, 4.0 times longer than central width, somewhat bowed, with simple setae medially, short sparse setae dorsally; ischium 0.5 times merus length, slightly expanded medially, with few simple setae medially; basis slightly smaller than ischium, with few simple setae medially; coxa with small ventral lobe with few short simple setae.

Second pereiopods (Fig. 12B–C) similar in form, unequal in length. Major right chela 1.8 times postorbital carapace length, palm smooth, compressed, without carinae, with few scattered simple short setae; fingers with few simple setae in distal part; dactylus 0.5 of palm length, 3.3 times longer than deep, with large broad flattened tooth with row denticles at almost halfway of cutting edge, distal part of cutting edge entire, tip strongly hooked; fixed finger 1.9 times as long as deep, with broad flattened tooth in proximal part, separated by shallow notch from acute triangular tooth at about distal third of cutting edge, distal part of cutting edge entire, straight, tip strongly hooked; carpus 0.4 of palm length, about as long as distal width, strongly tapering proximally; merus 1.4 times as long as carpus, 1.2 times longer than central width, distomedially excavate; ischium much shorter than merus, tapering proximally, with slightly protruded distomedial angle; basis and coxa without special features. Minor left cheliped with chela 1.2 times postorbital carapace length, dactylus slightly longer in relation to palm than in major chela; palm less swollen than in major chela.

**Figure 13.**
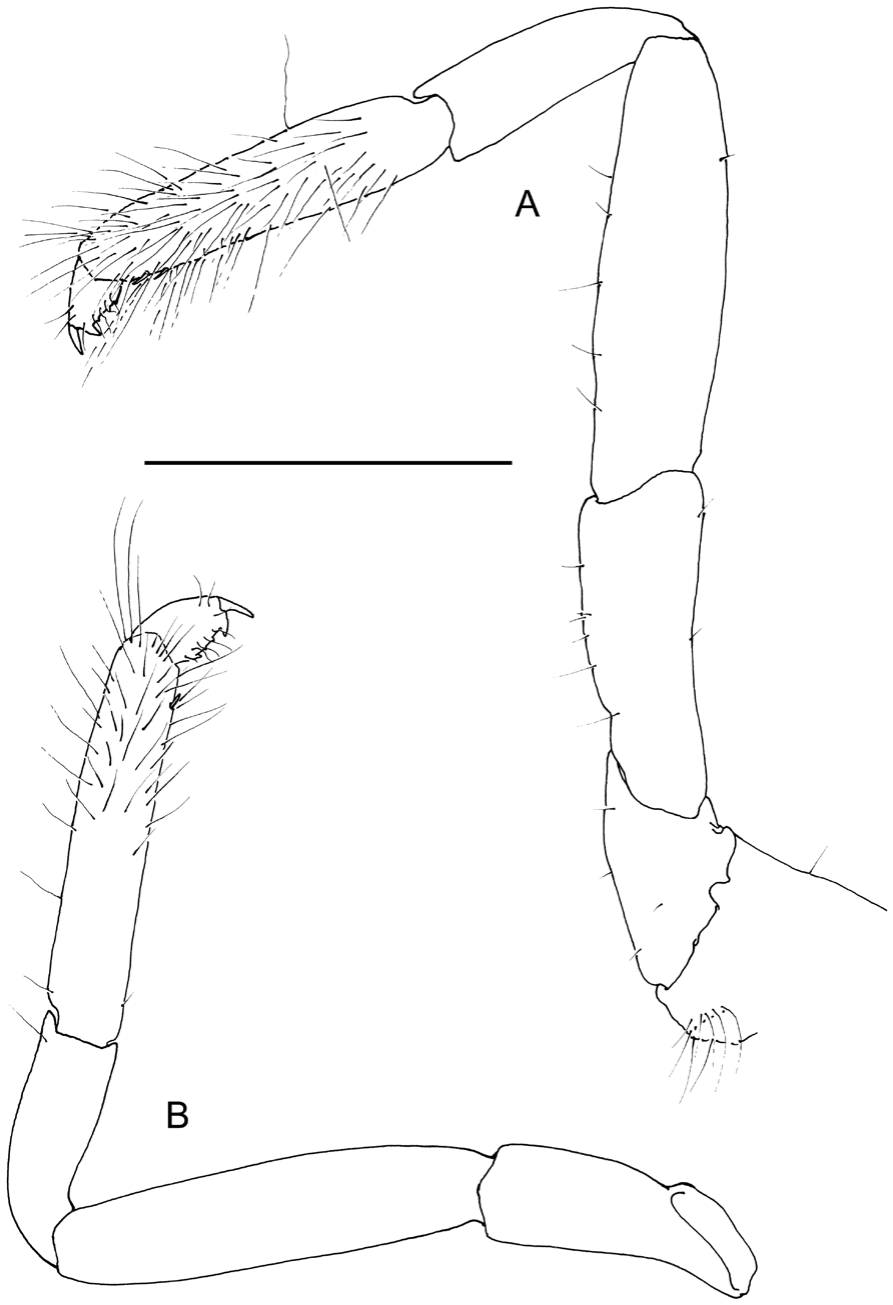
*Odontonia bagginsi* sp. n., ovigerous female PoCL 3.40 mm (MZB Cru 4733). **A** third pereiopod **B** fifth pereiopod. Scale bar: 1 mm.

**Figure 14.**
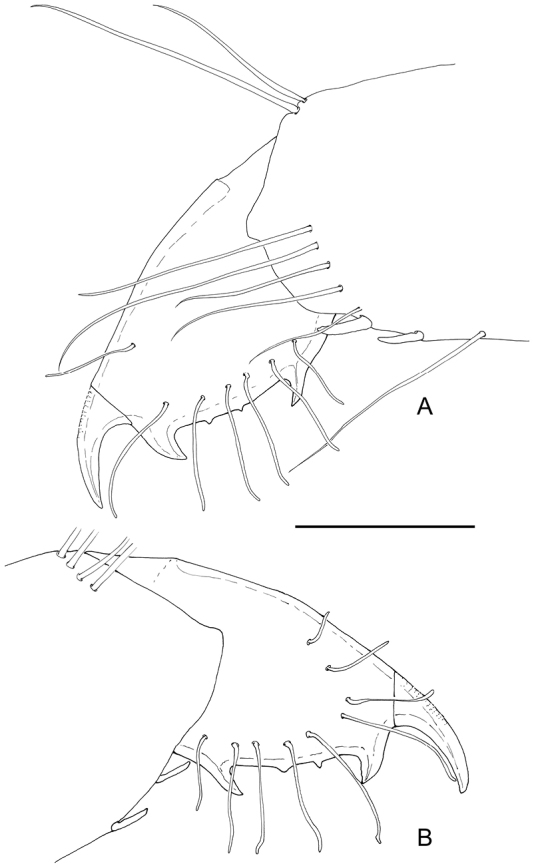
*Odontonia bagginsi* sp. n., ovigerous female PoCL 3.40 mm (MZB Cru 4733). **A** dactylus third pereiopod, medial view **B** dactylus third pereiopod, ventral view. Scale bar: 0.25 mm.

Ambulatory pereiopods short, stout. Dactylus of third pereiopod (Figs 13A, 14A–B, 16A, 17A) with corpus compressed, 1.8 times longer than proximal width, accessory tooth terminal, blunt, perpendicular to flexor margin, flexor margin with one large acute forward directed tooth at proximal third, with two small denticles in between proximal tooth and accessory tooth, with few simple setae at distolateral surface, with row of simple short setae along flexor margin; unguis longer than accessory tooth, acute, 0.43 of corpus length, without terminal scales, with faint proximal transverse grooves; propodus stout, compressed, 4.0 times length of dactylus, 4.0 times longer than deep, with minute lateral distoventral spine, and distal ventral spine, with many long simple setae on lateral margin; carpus 0.7 of propodus length, unarmed; merus 1.3 times propodus length, 3.7 times longer than central depth, unarmed; ischium 0.7 of merus length, slightly tapering proximally; basis and coxa without special features. Fourth and fifth (Fig. 13B) pereiopods similar.

First pleopod with endopod almost half as long as exopod, with plumose setae laterally and distally, with long simple setae distomedially.

Uropods, with short unarmed protopodite; exopod broad, 2.2 times longer than central width, lateral margin feebly convex, without distolateral tooth, with minute spinule distolaterally; endopod exceeding exopod, about as long as telson, 2.8 times longer than wide.

Ovigerous female with about 100 eggs of 0.05 mm in diameter.

###### Colour in life

**Figure 15.**
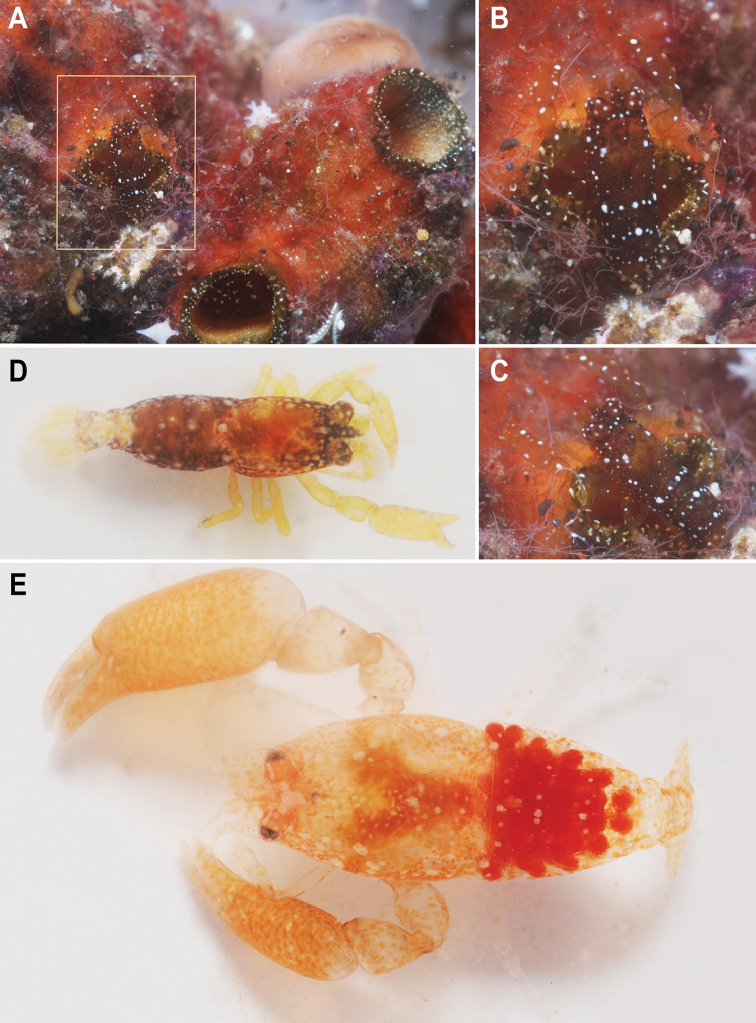
Colour patterns. **A–D** *Odontonia plurellicola* sp. n. (RMNH.CRUS.D.53554). **A–C** individuals inside *Plurella* sp. **D** outside host **E** *Odontonia bagginsi* sp. n. (MZB Cru 4733), outside host.

(Fig. 15E). Body and chelipeds generally semitransparent, with small red chromatophores and scattered larger white spots. Carapace with larger white chromatophores at base of rostrum and scattered in a bilaterally symmetrical pattern. Eyestalks reddish with some big dorsal white spots, cornea with white spots as well. Antennular peduncle and scaphocerite reddish. Ambulatory pereiopods translucent with white chromatophores at joints. Abdomen reddish with many small white chromatophores and large white spots dorsally and laterally at fixed distances. Tailfan with red and white chromatophores. Eggs dark red.

###### Host.

Solitary ascidian (A. Gittenberger Asc. 67).

###### Distribution.

Only known from its type locality at Tidore, Indonesia.

###### Etymology.

The species is named “bagginsi”, inspired by the famous Hobbit family name “Baggins” featured in the “The Hobbit” and “The Lord of the Rings” books. The fictional characters called “Hobbits” possess hairy feet comparable to this species.

###### Remarks.

The species bears resemblance to *O. sibogae* in its morphological characters. It differs in having a strongly developed ventral tooth on the rostrum; a strongly produced pterygostomial margin of the carapace; two pairs of dorsal spines on the telson and a broad upper lacinia of the maxillula. In addition, the pereiopods bear some notable differences: the segments of the first pereiopods are stouter and the cutting edge of the major cheliped bears a broad flattened tooth. The dactyli of the ambulatory pereiopods bear two denticles on the flexor margin. The unguis is devoid of distal scales. Most characteristic is the dense cover of simple setae on the propodi of the ambulatory pereiopods. Life colour patterns of the new species is similar to that of *O. sibogae* (see Levitt & Shenkar 2018: fig. 2B) with scattered white chromatophores of various sizes scattered over body and appendages. The new species has a reddish overall appearance while *O. sibogae* is paler.

### Phylogeny

The morphological phylogenetic analysis (Fig. 19), with *O. maldivensis* not yet synonymised with *O. rufopunctata*, indicates the sister position of *Odontonia plurellicola* sp. n. to *O. simplicipes* as well as the sister position of *O. bagginsi* to *O. sibogae*. *Odontonia maldivensis* and *O. rufopunctata* end up as sister species as well. The resolution in the basal part of the tree is low resulting in a polytomy.

The resulting tree from the incomplete COI dataset (Fig. 20) indicates the sister position of *O. bagginsi* to *O. sibogae* although support values are low. Bootstrap and posterior probability support values in the basal part of the tree are low in the morphological phylogenetic reconstruction.

The resulting tree of the incomplete 16S dataset (Fig. 21) is in line with the COI and morphological phylogenetic reconstructions. The basal branch within the *Odontonia* clade is not well supported. The 16S sequences of *O. maldivensis* and *O. rufopunctata* are similar (genetic distance between 0.012 and 0.092) and end up in the same clade. The genetic distance between the sister species *O. sibogae* and *O. katoi*, for instance, is 0.142.

### Hosts

Most *Odontonia* species live as endosymbionts in ascidian species in the order Stolidobranchia (Fig. 19). An exception is a single record of *O. sibogae* in the phlebobranch *Rhopalaea* (Fransen 2002). All other records of *O. sibogae* are from stolidobranch hosts (Fransen 2002). The other exception is *O. plurellicola* sp. n., which was found in the phlebobranch *Plurella* species.

## Discussion and conclusions

Both molecular and morphological phylogenetic analyses show good resolution in the distal part of the tree while basal support is low. The sister position of the species pair *O. sibogae* – *O. bagginsi* in the morphological tree is confirmed by the COI phylogeny. Low support in the basal nodes of the molecular phylogenies might be an effect of the chosen markers. More conservative markers might give better resolution in the basal part of the tree. However, the low support of the basal branches in the molecular trees and the basal polytomy in the morphological phylogeny could also be an indication of a rapid radiation over the host species as was shown for Caribbean sponge-dwelling snapping shrimps *Synalpheus* (Morrison et al. 2004).

The similar 16S sequences of *O. rufopunctata* and *O. maldivensis* as well as their highly similar morphology, indicate that these nominal species actually form one species. Differences indicated by Fransen (2006) are: 1) the ventral subdistal tooth of the rostrum being absent in *O. maldivensis* while present in *O. rufopunctata*, 2) the unguis of the ambulatory pereiopods of *O. maldivensis* (Fig. 18A) has a distal patch with more transverse rows of scales compared to *O. rufopunctata* (Fig. 18B), 3) the distal accessory tooth on the corpus of the dactylus is absent in *O. maldivensis* while present in *O. rufopunctata*, and 4) the dactyli of the second chelipeds have a pile of long simple setae on the dorsal surface in *O. maldivensis* while less setae are present in *O. rufopunctata*. In some specimens of *O. maldivensis*, however, a very small accessory tooth is present on the dactylus of the ambulatory legs (Fig. 17G). A striking character which was observed in both nominal species is the presence of simple short setae implanted in pits on the dorsal surface of the unguis of the ambulatory dactyli (Fig. 18C, D). It seems the morphological differences initially observed by Fransen (2006) might be mere intraspecific variation. *Odontonia maldivensis* Fransen, 2006 is therefore synonymised with *O. rufopunctata* Fransen, 2002.

**Figure 16.**
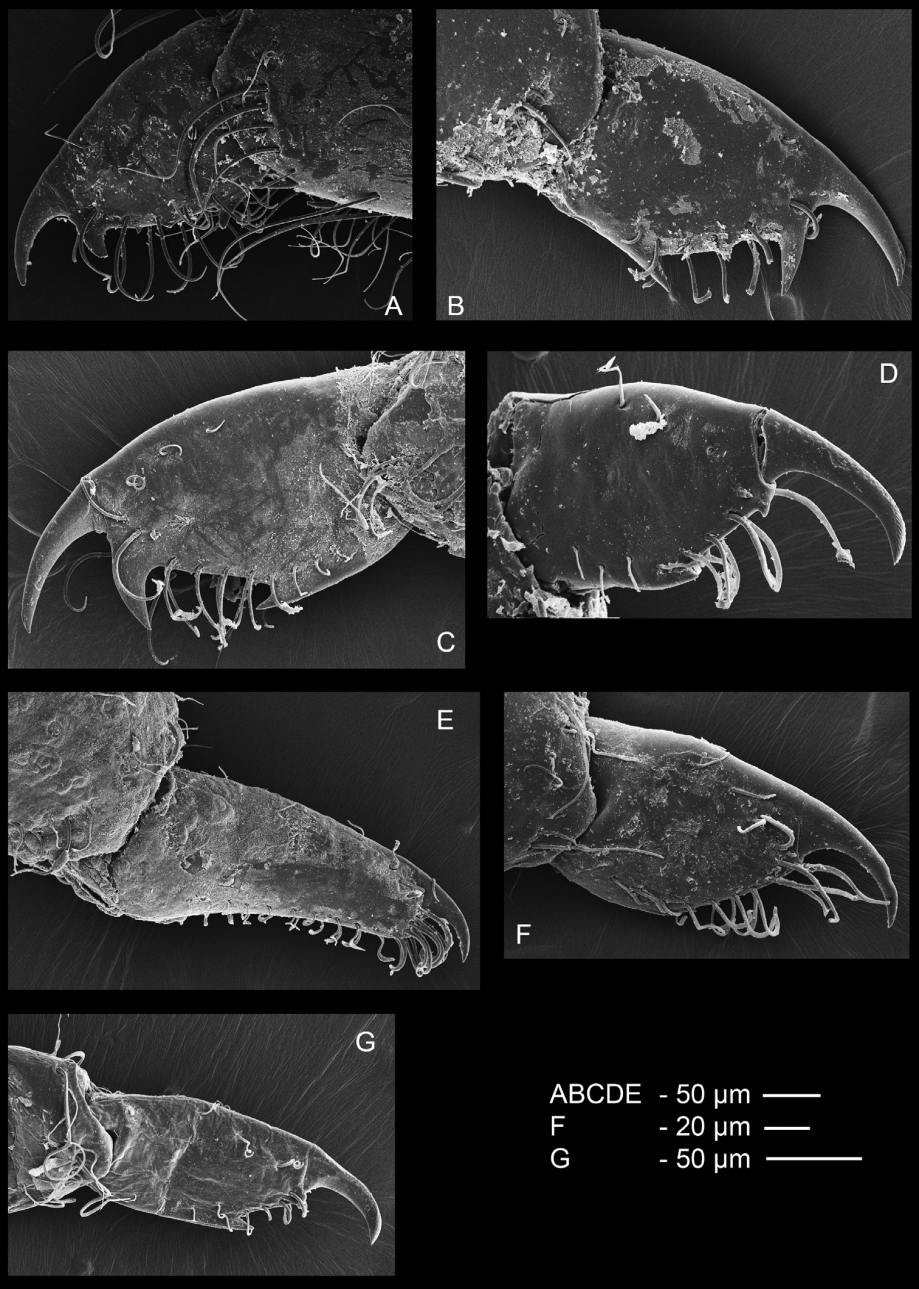
SEM photos dactylus third pereiopod. **A** *Odontonia bagginsi* sp. n. **B** *O. sibogae* (Bruce, 1972) **C** *O. katoi* (Kubo, 1940) **D** *O. rufopunctata* Fransen, 2002 **E** *O. seychellensis* Fransen, 2002 **F** *O. plurellicola* sp. n. **G** *O. maldivensis* Fransen, 2006.

**Figure 17.**
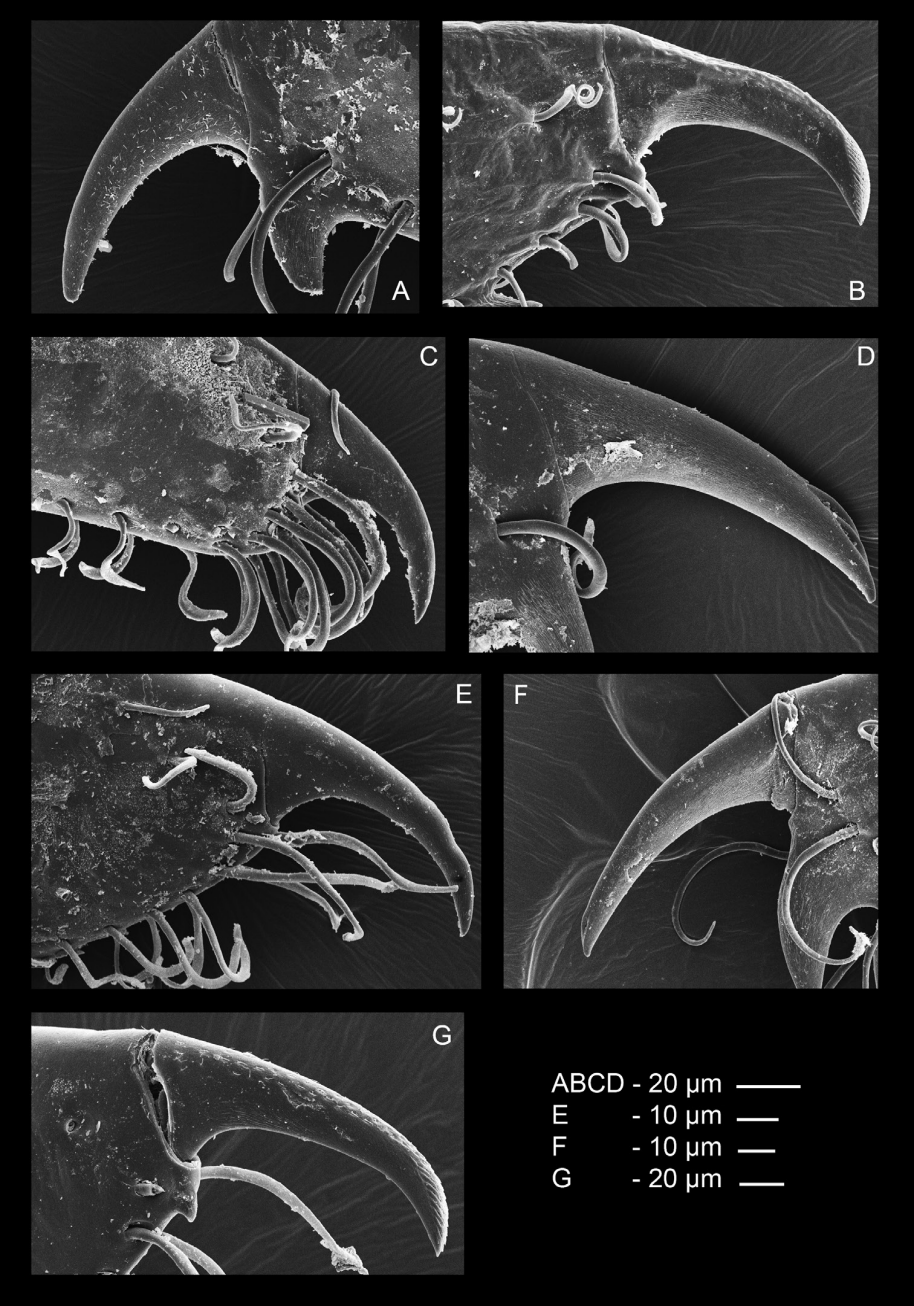
SEM photos unguis of third pereiopod. **A** *Odontonia bagginsi* sp. n. **B** *O. maldivensis* Fransen, 2006 **C** *O. seychellensis* Fransen, 2002 **D** *O. sibogae* (Bruce, 1972) **E** *O. plurellicola* sp. n. **F** *O. katoi* (Kubo, 1940) **G** *O. rufopunctata* Fransen, 2002.

**Figure 18.**
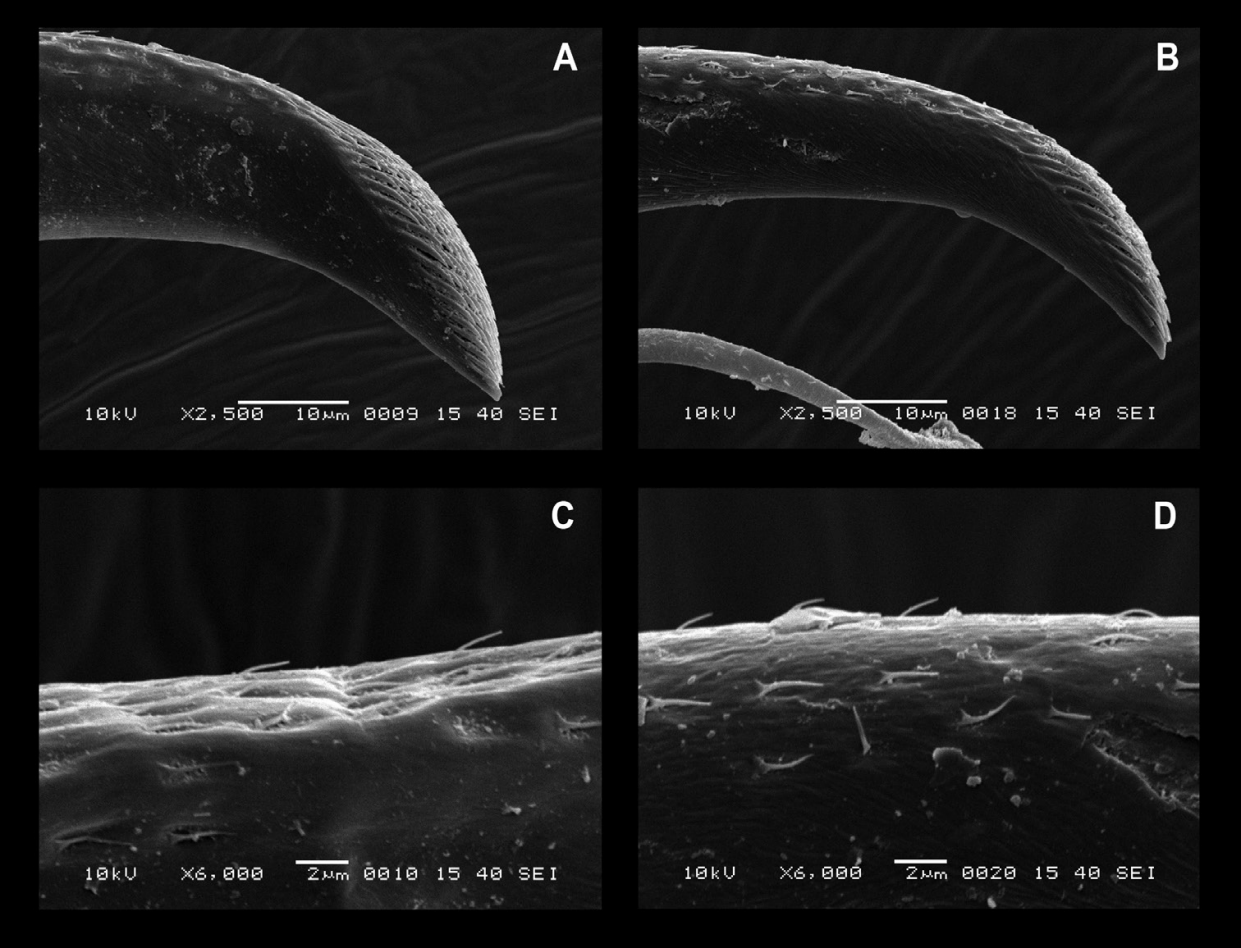
SEM photos details unguis. **A** *Odontonia maldivensis* Fransen, 2002, tip. **B** *O. rufopunctata* Fransen, 2002, tip **C** *O. maldivensis*, dorsal surface **D** *O. rufopunctata*, dorsal surface.

Several *Odontonia* species (*O. simplicipes*, *O. compacta*, and *O. bagginsi* sp. n.) are only known from a single or few type specimens. Therefore, intraspecific morphological variation is not known and molecular data are not available (except for *O. bagginsi* sp. n.) which hampers the present phylogenetic analyses. When more material of these rare species comes available a more comprehensive phylogenetic analysis can be performed.

**Figure 19.**
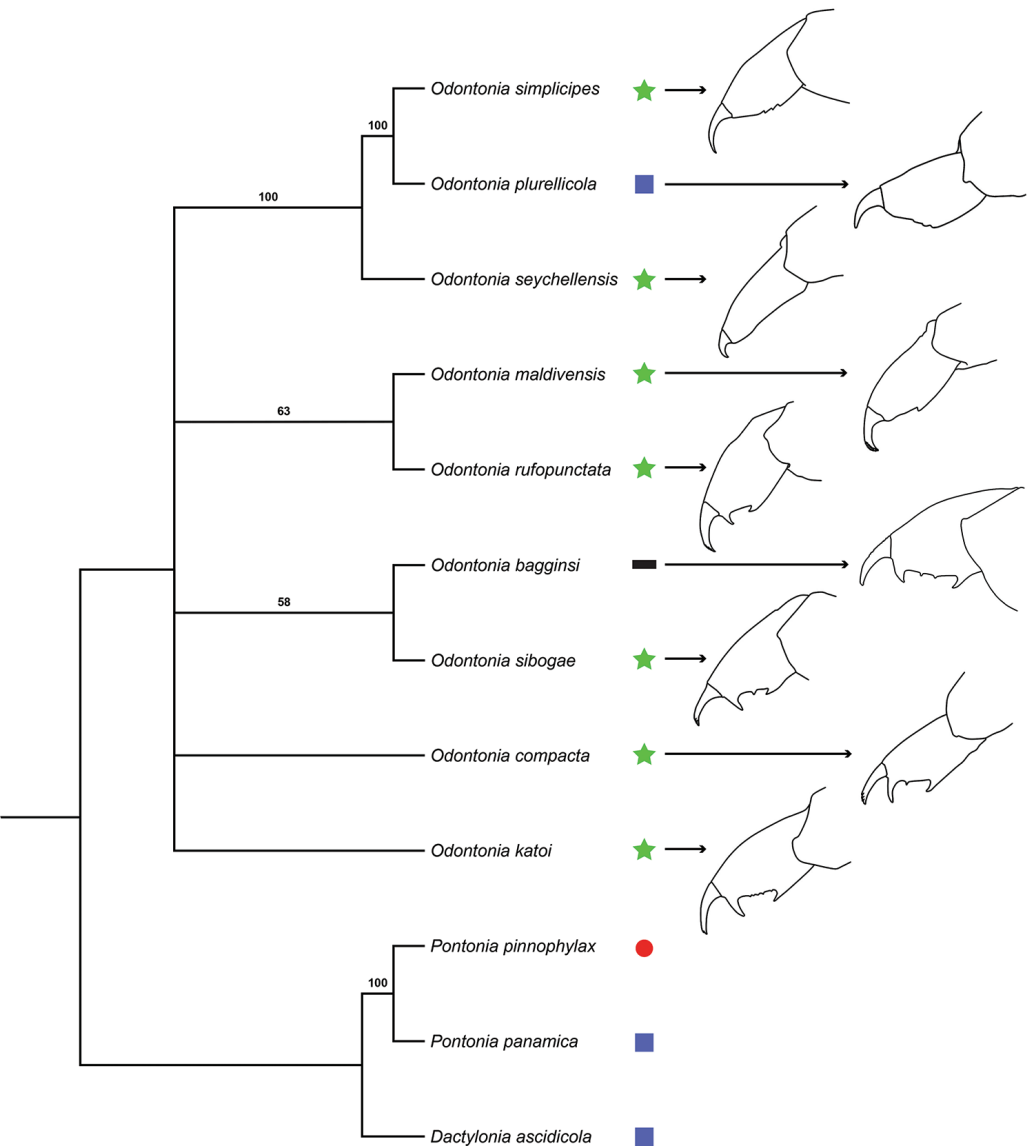
Phylogenetic analysis based on morphological dataset (Table 2). 50% majority-rule tree, CI=0.58. Dactyli of third pereiopods shown for *Odontonia* species, and host group (★: Stolidobranchia, ■: Phlebobranchia, ●: Mollusca, Bivalvia, ▂: solitary ascidian).

Most *Odontonia* species live as endosymbionts in ascidian species in the order Stolidobranchia (Fig. 19) (Fransen 2002). Taxa in this order are characterised by having folded internal structures (branchial sacs). These folded structures probably limit the amount of space the shrimp have inside the ascidian, which could be an explanation for the compact and spineless body of *Odontonia* species. The ascidian associated outgroup species have members of the order Phlebobranchia as their host. The phlebobranch ascidians do not have the stolidobranchial folded branchial sac, which might explain the larger and somewhat less smooth, rounded bodies of these outgroup species. Members of the genus *Plurella* are colonial ascidians, but they do not share a common branchial sac. Shrimp living inside *Plurella* would not be able to move internally from ascidian to ascidian. From the phylogenetic reconstruction of *Odontonia* it can be deduced that *O. plurellicola* switched from a stolidobranch host to a phlebobranch host. Species of *Plurella* have also been recorded as host for the palaemonid shrimp *Dactylonia holthuisi*.

**Figure 20.**
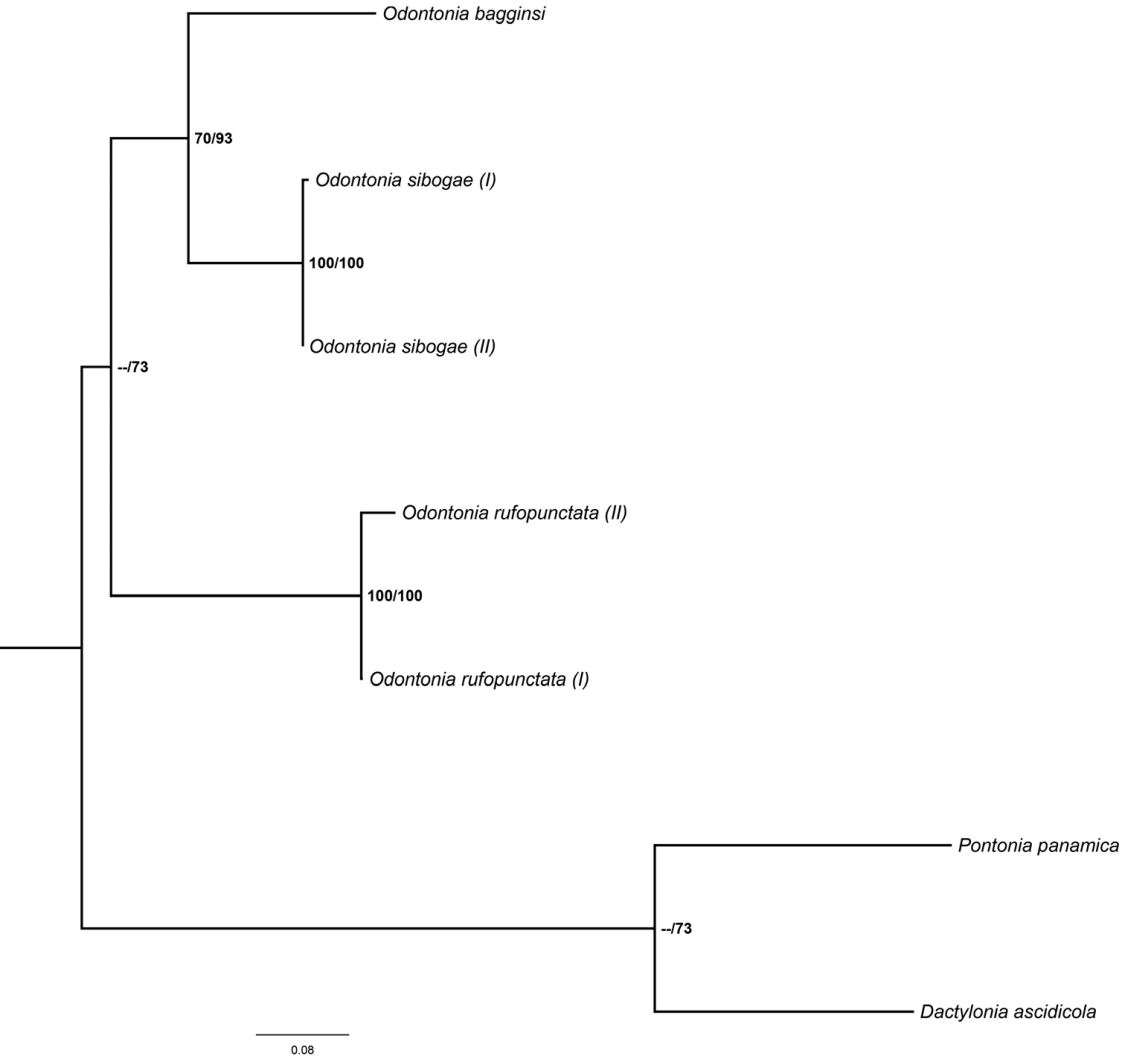
Phylogenetic analysis based on the COI barcoding gene of a subset of *Odontonia* species (Table 1). Maximum likelihood tree with Bootstrap values and Bayesian posterior possibility values respectively.

**Figure 21.**
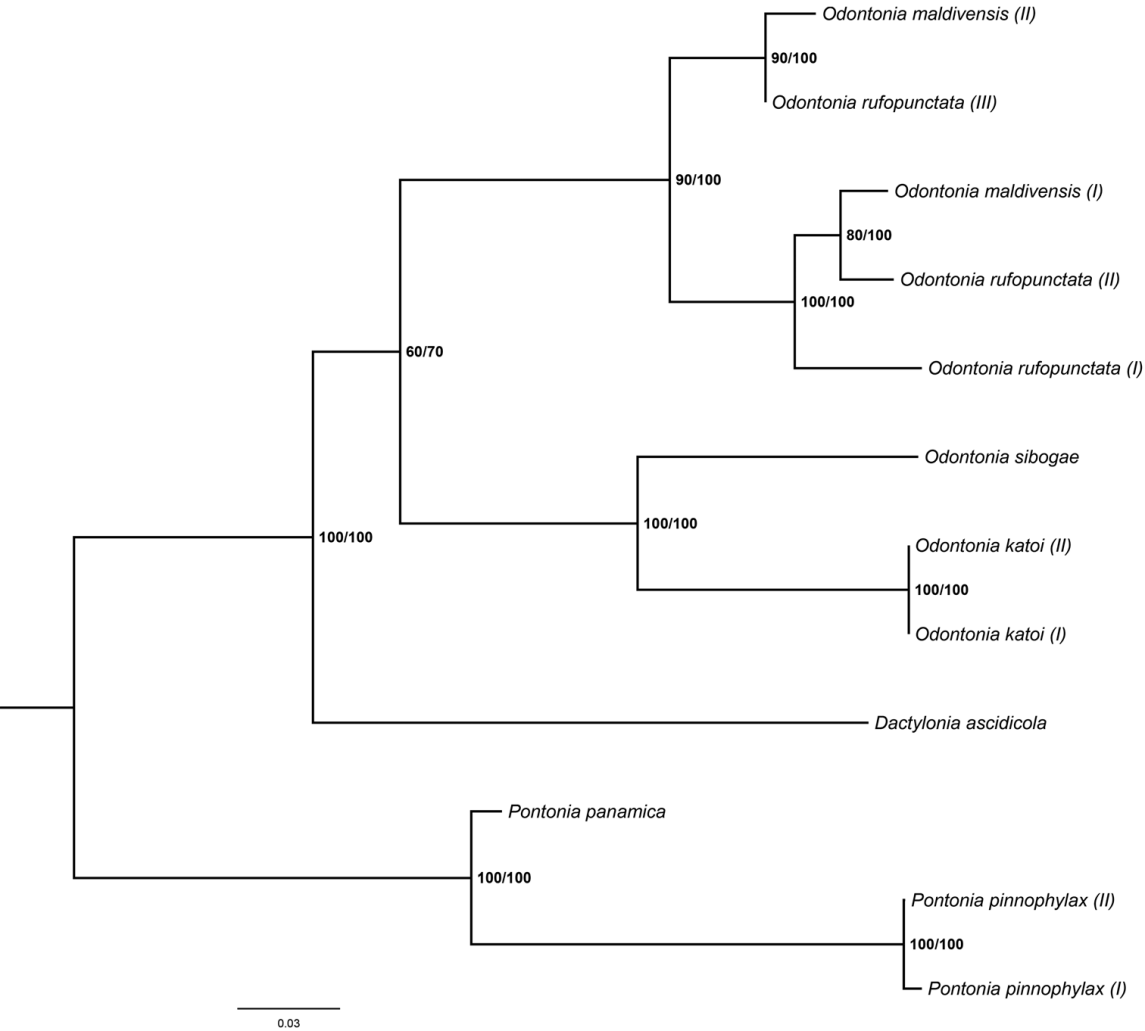
Phylogenetic analysis based on the 16S mitochondrial ribosomal gene of a subset of *Odontonia* species (Table I). Maximum likelihood tree with Bootstrap values (first value) and Bayesian posterior possibility values (second value).

### Key to the species of the genus *Odontonia*

**Table:** 

1	Dactylus of ambulatory pereiopods without proximal teeth on flexor margin or with row of few shallow, forward directed teeth; unguis glabrous	**2**
–	Dactylus of ambulatory pereiopods with single large forward directed proximal tooth on flexor margin; distodorsal scales on unguis (except in *O. bagginsi*)	**4**
2	Dactylus of ambulatory pereiopods with row of few shallow, forward directed teeth on flexor margin	**3**
–	Dactylus of ambulatory pereiopods with flexor margin entire	***O. seychellensis***
3	Rostrum with distal ventral tooth; distolateral tooth of antennular basal segment slightly exceeding distal margin of segment	***O. plurellicola* sp. n.**
–	Rostrum without distal ventral tooth; distolateral tooth of antennular basal segment reaching distal margin of intermediate segment	***O. simplicipes***
4	Unguis of dactylus of ambulatory pereiopods without or with few distal scales (not more than 5); small denticles between accessory and proximalmost tooth	**5**
–	Unguis of dactylus of ambulatory pereiopods with patch with many distal scales; no denticles between accessory and proximalmost tooth	***O. rufopunctata***
5	Rostrum not overreaching antennular peduncle	**6**
–	Rostrum overreaching antennular peduncle	**7**
6	Telson with 2 pairs of dorsal spines; unguis without scales; propodi of ambulatory pereiopods with many long simple setae	***O. bagginsi* sp. n.**
–	Telson with 5 pairs of dorsal spines; unguis with few distodorsal scales; propodi of ambulatory pereiopods almost devoid of setae	***O. sibogae***
7	Unguis of dactylus of ambulatory pereiopods with two distal scales, and one denticle between accessory and proximalmost tooth	***O. compacta***
–	Unguis of dactylus of ambulatory pereiopods with one distal scale, and (with or without) five small denticles between accessory and proximalmost tooth	***O. katoi***

## Supplementary Material

XML Treatment for Odontonia

XML Treatment for Odontonia plurellicola

XML Treatment for Odontonia bagginsi

## Figures and Tables

**Figure A1.**
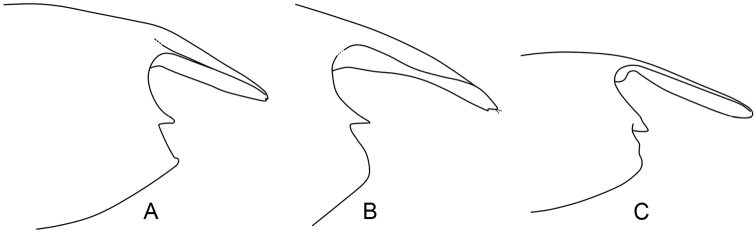
Variety in rostrum morphology and anterolateral angle variety in three *Odontonia* species. Note that A and B have a terminal tooth on the rostrum, while C has no teeth. **A** *O. katoi* (Kubo, 1940) **B** *O. rufopunctata* Fransen, 2002 **C** *O. simplicipes* (Bruce, 1996).

**Figure A2.**
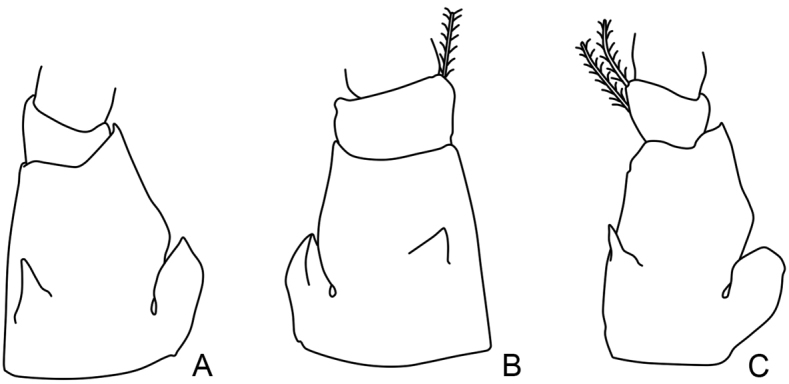
Variety in antennular peduncle. Note the well-developed ventromedial tooth in **A, B, C** and the amount of plumose hairs in **B** and **C**. **A** *O. katoi* (Kubo, 1940) **B** *O. plurellicola* sp. n. **C** *O. sibogae* (Bruce, 1972).

**Figure A3.**
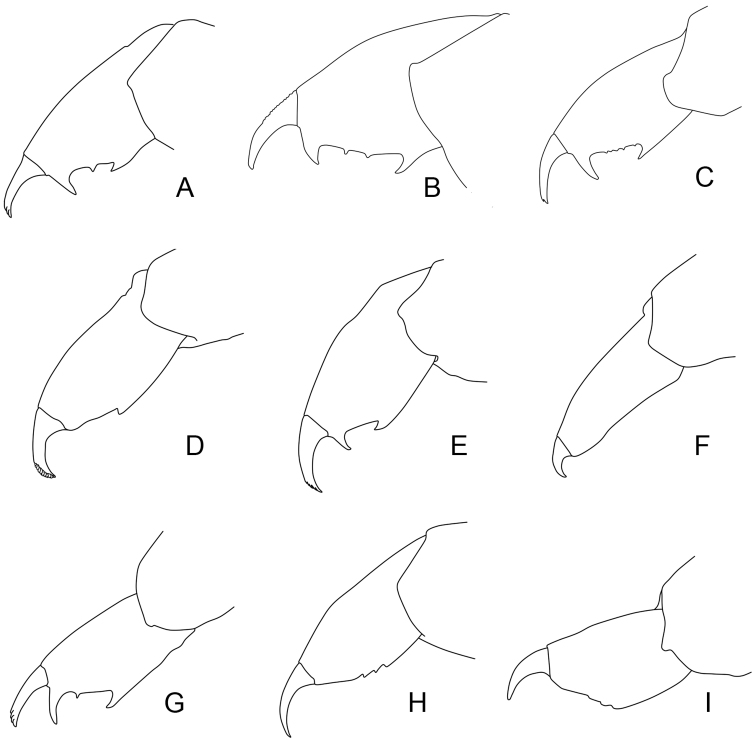
Variety in dactylar morphology of all currently known *Odontonia* species (setae omitted). Note the amount of distal scales, presence of an additional and accessory tooth, size of the dactylus, and the presence of denticles. **A** *O. compacta* (Bruce, 1996) **B** *O. bagginsi* sp. n. **C** *O. katoi* (Kubo, 1940) **D** *O. maldivensis* Fransen, 2006 **E** *O. rufopunctata* Fransen, 2002 **F** *O. seychellensis* Fransen, 2002 **G** *O. sibogae* (Bruce, 1972) **H** *O. simplicipes* (Bruce, 1996) **I** *O. plurellicola* sp. n.

## Appendix 1

### Character state analysis

#### Rostrum

1. Ventral teeth: 0, absent; 1, one subdistal; 2, one apical. Character states 0 and 1 are found in the outgroups. An apical tooth (often accompanied by some distal setae) is found in most *Odontonia* species, while a subdistal tooth is found only in the *Pontonia* members of the outgroup. No tooth is found in the outgroup *Dactylonia ascidicola*, *in O. simplicipes* and *O. maldivensis*. (Fig. A1).

2. Length of rostrum: 0, falling short of antennular peduncle; 1, around the same length as antennular peduncle; 2, overreaching antennular peduncle. A small rostrum is found in all outgroups, while in most *Odontonia* species the rostrum is overreaching, or as long as, the antennular peduncle.

3. Relative size rostrum to post-orbital carapace length: 0, around 0.15 to 0.45 times the PoCL; 1, around 0.75 times the PoCL. All outgroup species, as well as most *Odontonia* species possess a small rostrum-PoCL ratio, while only *O. simplicipes* has a rostrum-PoCL ratio of 0.75.

4. Relative size rostrum to hemispherical diameter of cornea: 0, around 4.0 times the cornea size; 1, around 2.5 to 3.0 times the cornea size; 2, around 2.0 times the cornea size. A rostrum-cornea ratio of 4.0 is found in the outgroup species *Pontonia pinnophylax* (Otto, 1821) and *P. panamica*, while most *Odontonia* species and the other outgroup species have a smaller ratio.

#### Carapace

5. Anterolateral angle: 0, absent; 1, present. No angle is found in the outgroup species, as well as in *O. rufopunctata* Fransen, 2002 and *O. bagginsi* sp. n. (Fig. A1).

#### Antennulae

6. Ventromedial tooth basal segment: 0, small; 1, strongly developed; A strongly developed tooth is found in almost all *Odontonia* species (hence the name of the genus), while a small tooth is found in the three outgroup species, and in *O. simplicipes* (Fig. A2).

7. Dimensions of intermediate segment: 0, about as long as wide or slightly broader than long; 1, about twice as long as wide. Most *Odontonia* species share character state 0 with the outgroups. *Odontonia plurellicola* sp. n., *O. simplicipes*, and *O. seychellensis* Fransen, 2002 have a longer intermediate segment. The character is treated ordered in the analysis.

8. Plumose setae on median margin of intermediate segment: 0, two or more setae; 1, one setae; 2, no setae. The specific setae are absent in *Pontonia panamica*, as well as in *O. katoi* (Kubo, 1940) and *O. simplicipes*. The other two outgroup species have two or more setae, and most *Odontonia* species share this treat (Fig. A2).

#### Mouthparts

9. Maxilla: 0, upper lacinia and lower lacinia both well developed; 1, upper lacinia well-developed, lower reduced; 2, both lacinia almost completely fused. *Dactylonia ascidicola* is the only species in the analysis with an uneven development of the two lacinia. The other outgroups share both developed lacinia with almost all *Odontonia* species. *Odontonia rufopunctata* has no more distinguishable lacinia, both are almost completely fused.

10. First maxilliped; amount of distal setae on exopod: 0, eight or more setae; 1, five or six setae; 2, four setae. This character is treated as ordered; it is believed that the number of setae decreases in the exopods of *Odontonia* species due to their symbiotic lifestyle in ascidians.

11. Second maxilliped; angle in median margin of basis: 0, absent; 1, distinct angle. Character state 1 is found in two outgroup species, except for *Pontonia panamica*. Most *Odontonia* species do have an angle in the basis, with the exception of *O. maldivensis* and *O. rufopunctata*.

12. Second maxilliped; amount of distal setae on exopod: 0, eight or more setae; 1, five or six setae; 2, four setae. This character is treated as ordered (see character 10).

13. Third maxilliped; amount of distal setae on exopod: 0, eight or more setae; 1, five or six setae; 2, four setae. This character is treated as ordered (see character 10).

#### Pereiopods

14. First pereiopod; ratio merus/carpus: 0, merus as long as carpus; 1, carpus shorter than merus. Character state 1 is present in outgroup species *Dactylonia ascidicola*, *O. rufopunctata* and *O. bagginsi* sp. n.

15. Third pereiopod; dactylus; teeth on flexor margin: 0, teeth absent; 1, dactylus with simple teeth; 2, dactylus with minutely spinulate blunt tubercles. Character state 2 is only found in *Dactylonia ascidicola*, and character state 0 is found in the two *Pontonia* outgroup species, and in *O. seychellensis* (Fig. A3).

16. Third pereiopod; dactylus; teeth on flexor margin: 0, teeth absent; 1, teeth similar, increasing in size; 2, proximalmost tooth strong, large, directed forward. The teeth of *Dactylonia ascidicola* are very different from the *Odontonia* and *Pontonia* species, hence character state 1. All *Odontonia* with teeth on their flexor margin have one large proximalmost tooth, with or without more teeth following (Fig. A3).

17. Third pereiopod; dactylus, unguis: 0, distal scales absent; 1, unguis with fewer than five distal scales; 2, unguis with more than five distal scales. Scales are absent in the two *Pontonia* outgroup species, as well as in *O. seychellensis*, *O. simplicipes*, *O. plurellicola* sp. n., and *O. bagginsi* sp. n. The species *O. maldivensis* and *O. rufopunctata* have more than five distal scales (Fig. A3). This character is treated as ordered, the amount of distal scales is believed to increase the further a species is located from the outgroups.

18. Third pereiopod; dactylus; dimensions of corpus: 0, more than 2.0 times as long as broad; 1, less than 2.0 times as long as broad. *Dactylonia ascidicola*, *Pontonia panamica* and *O. seychellensis* all share a dactylus that is more than 2.0 times as long as broad (Fig. A3).

19. Third pereiopod; dactylus; accessory tooth: 0, present; 1, absent. Most *Odontonia* species, as well as the outgroup species, possess an accessory tooth on the distal flexor margin. *Odontonia* seychellensis, *O. simplicipes*, *O. maldivensis* and *O. plurellicola* sp. n. all have no accessory tooth (Fig. A3).

20. Third pereiopod; dactylus; additional forward-pointing teeth distal from proximalmost tooth: 0, absent; 1, two additional forward-pointing teeth. Only *O. simplicipes* and *O. plurellicola* sp. n. possess the two additional teeth (Fig. A3).

21. Third pereiopod; dactylus; denticles between accessory and proximalmost tooth: 0, absent; 1, one, two or five denticles in flexor margin. *Odontonia sibogae* and *O. compacta* are the only ones with one denticle. *Odontonia bagginsi* sp. n. has two denticles. *Odontonia katoi* is the only species with accounts of there being a row of five denticles on the flexor margin, but often these denticles are not visible or not present. The outgroup *Dactylonia ascidicola* has broad denticles considered not homologous with the ones from *Odontonia* (Fig. A3). This character is treated as ordered.

#### Telson

22. Position of distal pair of dorsal spines over telson length: 0, at about 1/2; 1, at about 2/3; 2, in distal 1/4. Character state 0 is present in *Dactylonia ascidicola* and in *O. bagginsi* sp. n. The species *O. compacta* has the distal spines implanted in the most distal 1/4th of the telson length. In *O. sibogae* there are more than four spines implanted in the dorsal telson side. It is not sure which spines are homologous with the four spines in other species, hence the “?” in the dataset. This character is treated as ordered; it is believed the spines migrate more distally in the evolution of the shrimp.

#### Length of post-orbital carapace length

23. Maximum PoCL of male specimens: 0, PoCL longer than 5.5 mm; 1, PoCL between 5.5 and 2.0 mm; 2, between 2.0 and 0.5 mm. Depending on the availability of the specimens a “?” may be given to missing character states.

24. Maximum length of female specimens: 0, PoCL longer than 5.5 mm; 1, between 5.5 and 2.0 mm; 2, between 2.0 and 0.5 mm. Depending on the availability of the specimens a “?” may be given to missing character states.
